# The glycerol-3-phosphate dehydrogenases GpsA and GlpD constitute the oxidoreductive metabolic linchpin for Lyme disease spirochete host infectivity and persistence in the tick

**DOI:** 10.1371/journal.ppat.1010385

**Published:** 2022-03-07

**Authors:** Dan Drecktrah, Laura S. Hall, Bethany Crouse, Benjamin Schwarz, Crystal Richards, Eric Bohrnsen, Michael Wulf, Bonnie Long, Jessica Bailey, Frank Gherardini, Catharine M. Bosio, Meghan C. Lybecker, D. Scott Samuels

**Affiliations:** 1 Division of Biological Sciences, University of Montana, Missoula, Montana, United States of America; 2 Laboratory of Bacteriology, National Institute of Allergy and Infectious Diseases, National Institutes of Health, Hamilton, Montana, United States of America; 3 Department of Biology, University of Colorado, Colorado Springs, Colorado, United States of America; 4 Center for Biomolecular Structure and Dynamics, University of Montana, Missoula, Montana, United States of America; Medical College of Wisconsin, UNITED STATES

## Abstract

We have identified GpsA, a predicted glycerol-3-phosphate dehydrogenase, as a virulence factor in the Lyme disease spirochete *Borrelia* (*Borreliella*) *burgdorferi*: GpsA is essential for murine infection and crucial for persistence of the spirochete in the tick. *B*. *burgdorferi* has a limited biosynthetic and metabolic capacity; the linchpin connecting central carbohydrate and lipid metabolism is at the interconversion of glycerol-3-phosphate and dihydroxyacetone phosphate, catalyzed by GpsA and another glycerol-3-phosphate dehydrogenase, GlpD. Using a broad metabolomics approach, we found that GpsA serves as a dominant regulator of NADH and glycerol-3-phosphate levels *in vitro*, metabolic intermediates that reflect the cellular redox potential and serve as a precursor for lipid and lipoprotein biosynthesis, respectively. Additionally, GpsA was required for survival under nutrient stress, regulated overall reductase activity and controlled *B*. *burgdorferi* morphology *in vitro*. Furthermore, during *in vitro* nutrient stress, both glycerol and *N*-acetylglucosamine were bactericidal to *B*. *burgdorferi* in a GlpD-dependent manner. This study is also the first to identify a suppressor mutation in *B*. *burgdorferi*: a *glpD* deletion restored the wild-type phenotype to the pleiotropic *gpsA* mutant, including murine infectivity by needle inoculation at high doses, survival under nutrient stress, morphological changes and the metabolic imbalance of NADH and glycerol-3-phosphate. These results illustrate how basic metabolic functions that are dispensable for *in vitro* growth can be essential for *in vivo* infectivity of *B*. *burgdorferi* and may serve as attractive therapeutic targets.

## Author summary

Lyme disease (borreliosis) is the most common tick-borne disease in the Northern hemisphere and its prevalence is increasing. *Borrelia burgdorferi*, the etiological agent of Lyme disease, is an enzootic pathogen that alternates between a tick vector and vertebrate host. Humans are considered an incidental host after transmission of *B*. *burgdorferi* following the bite of an infected tick. The mechanisms by which *B*. *burgdorferi* persists in the Ixodid tick, transmits to a vertebrate host and establishes infection are not well understood. Therefore, identifying virulence factors and uncovering the pathogenic strategies in the spirochete remain important to address the public health concerns of Lyme disease. In this study, we identify an enzyme involved in three-carbon metabolism, GpsA, as a new virulence factor with an effect on persistence in ticks. GpsA and GlpD, another enzyme, constitute a bidirectional metabolic node connecting lipid biosynthesis and glycolysis, which serves as the linchpin for regulating carbon utilization for *B*. *burgdorferi* throughout its enzootic cycle. Disruption of this node causes a lethal metabolic imbalance revealing a potential therapeutic target for the treatment of Lyme disease.

## Introduction

Lyme disease is the most prevalent arthropod-borne infection in North America with an estimated 476,000 cases annually in the United States [1]. *Borrelia* (*Borreliella*) *burgdorferi*, the enzootic spirochete that causes Lyme disease [2–4], is maintained in nature by cycling between *Ixodes* ticks and a vertebrate host reservoir, primarily white-footed mice; the bacterium is neither free-living nor transovarially transmitted by the female ticks to oocytes. The reduced genome of *B*. *burgdorferi* reflects the constraints of host dependence where numerous biosynthetic and energy-producing metabolic pathways have been lost, including amino acid synthesis, nucleotide synthesis, fatty acid synthesis, the citric acid cycle, and the electron transport chain [5,6]. Thus, *B*. *burgdorferi* has evolved into an unabashed scavenger of amino acids, nucleosides, peptides and various carbon sources including glucose, *N*-acetylglucosamine (GlcNAc) and glycerol. The metabolic capacity retained by *B*. *burgdorferi* to flourish in the disparate environments of the vertebrate host and tick vector is important to understand as these strategies, in the absence of any identified toxins or secreted effectors, determine survival of the spirochete and thus the pathogenesis of Lyme disease.

**Fig 1:**
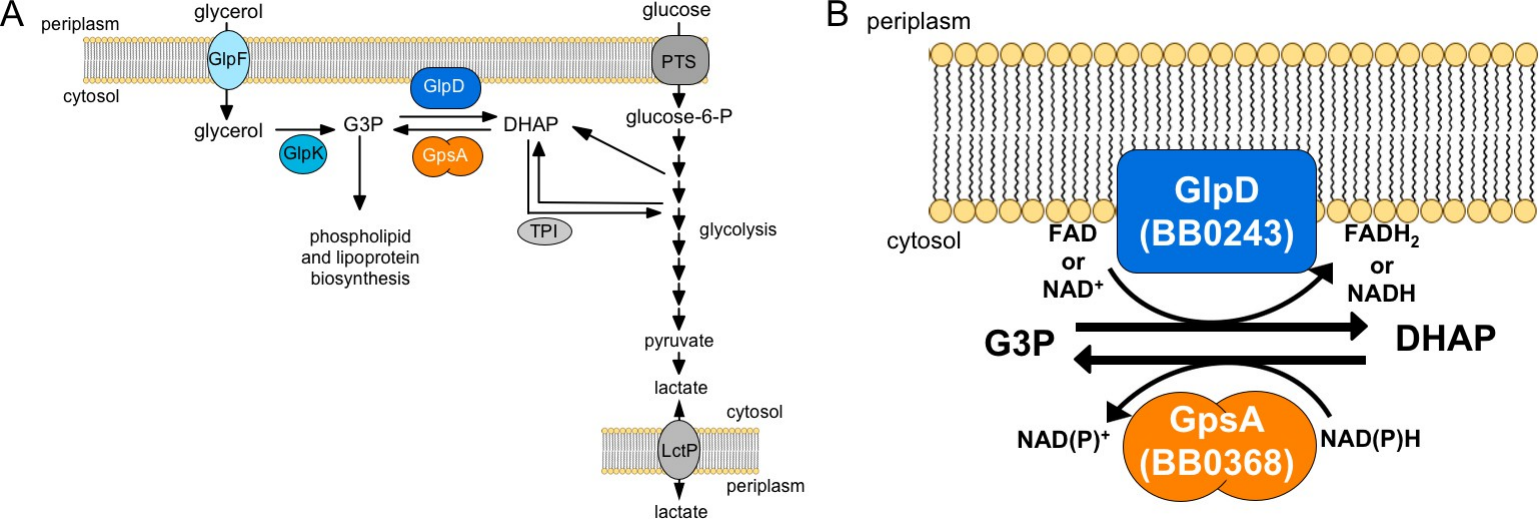
The redox linchpin connecting three-carbon (glycerol) metabolism and six-carbon (glycolysis) metabolism in *B*. *burgdorferi*. (A) Schematic overview of the intersection of glycerol metabolism and glycolysis, including the conversion of pyruvate to lactate and the use of glycerol-3-phosphate (G3P) for lipid and lipoprotein biosynthesis. Dihydroxyacetone phosphate (DHAP); glycerol uptake facilitator (GlpF, BB0240); glycerol kinase (GlpK, BB0241); glycerol-3-phosphate dehydrogenase (GlpD, BB0243); glycerol-3-phosphate dehydrogenase (GpsA, BB0368); triose phosphate isomerase (TPI); phosphotransferase systems (PTS); lactate permease (LctP). (B) This redox junction consists of two predicted glycerol-3-phosphate dehydrogenases, GlpD and GpsA. GlpD putatively oxidizes G3P to DHAP, while reducing flavin adenine dinucleotide (FAD) or NAD^+^, to feed glycerol into glycolysis. GpsA reduces DHAP to G3P, using the reducing power of NAD(P)H, to provide carbohydrates for lipoproteins and glycerophospholipids.

Available carbon sources are a dynamic determinant of *B*. *burgdorferi* persistence and transit through the enzootic cycle [7,8]. Glucose is likely the preferred carbon source of *B*. *burgdorferi* in the vertebrate host and initially during tick feeding, but other carbohydrates can support growth and have a role during the enzootic cycle [9–11]. In particular, glycerol becomes important for *B*. *burgdorferi* persistence in the tick as glucose levels decrease when the blood meal is consumed by the tick and its microbiome. This importance is exemplified by the finding that *B*. *burgdorferi* mutants unable to utilize glycerol for glycolysis are significantly compromised for persistence in the tick, yet remain infectious in the vertebrate host [12–14]. Glycerol also supports *B*. *burgdorferi* growth *in vitro*, particularly at 23°C, the temperature often used to mimic tick-like conditions [12,13,15]. Glycerol enters the cell through the glycerol uptake facilitator GlpF, is converted to glycerol 3-phosphate (G3P) by the glycerol kinase GlpK and either is shuttled to glycolysis via conversion to dihydroxyacetone phosphate (DHAP) by the glycerol-3-phosphate dehydrogenase (G3PDH) GlpD or serves as the three-carbon backbone for lipid and lipoprotein biosynthesis (Fig 1) [5–7]. The *glp* operon, consisting of the *glpF*, *glpK* and *glpD* genes, along with *bb0242*, is controlled by a diverse repertoire of regulatory mechanisms [16]. Gene expression is induced during nutrient stress by the stringent response mediated by Rel_Bbu_, (p)ppGpp and the effector protein DksA [15,17,18], by the response regulator Rrp1, which produces c-di-GMP [13,19], at 23°C [20], by glycerol [13,15], and in the tick [12]. RpoS and BadR both repress levels of *glp* operon transcripts, although BadR likely exerts its influence through RpoS [21,22]. Additionally, the c-di-GMP effector protein PlzA can either positively or negatively affect *glp* operon expression depending on its c-di-GMP-binding state [23]. These regulatory pathways targeting the *glp* operon represent the best understood strategies that *B*. *burgdorferi* uses to persist in the tick and illustrate the central role of glycerol regulation in this phase of the enzootic cycle. Other genes involved in carbohydrate utilization, such as *malQ* and *chbC*, are not required for *B*. *burgdorferi* in either its vertebrate host or tick vector [10,11].

GlpD shuttles G3P towards glycolysis via DHAP to provide energy while the reverse reaction, reducing DHAP to G3P mediated by another predicted G3PDH, GpsA, connects carbohydrates entering glycolysis to lipid and lipoprotein biosynthesis (Fig 1) [5–7]. In the absence of glycerol, GpsA, using carbohydrates from glycolysis, serves as the only known pathway to provide G3P for lipid and lipoprotein biosynthesis as *B*. *burgdorferi* cannot import this phosphorylated sugar alcohol or salvage glycerolipids [24]. *B*. *burgdorferi* must coordinate the activities of GlpD and GpsA to efficiently balance carbon sources and redox cofactors, such as NADH, and respond to the physiological requirements of the spirochete. Thus, the GlpD/GpsA metabolic node regulates carbon flow between lipid biosynthesis and glycolysis in response to the phase of the enzootic cycle as indicated by the available carbon sources.

In this study we examine the *in vivo* role of the GpsA/GlpD metabolic node in murine infectivity and tick persistence in an animal model of Lyme disease. Additionally, we molecularly dissect the contributions and interactions *in vitro* of these two G3PDHs in spirochete survival and morphology as well as broad metabolic regulation using a metabolomics approach.

## Results

*B*. *burgdorferi* has a reduced genome resulting in limited metabolic capacity where the only identified connection of glycerol metabolism and lipid biosynthesis to glycolytic energy production is the bidirectional oxidoreductase node mediated by the opposing actions of a pair of G3PDHs GlpD and GpsA (Fig 1) [5–7]. Based on sequence homology, GlpD is thought to oxidize G3P to DHAP and concomitantly reduce either NAD^+^ or FAD. GpsA is predicted to catalyze the reverse reaction to reduce DHAP to G3P using the reducing power of NADH or NADPH. Pappas et al., 2011 [12] and He et al., 2011 [13] have shown that GlpD and the *glp* operon, respectively, are important for *B*. *burgdorferi* growth on glycerol and for persistence in the tick, but dispensable for murine infectivity. The function of GpsA either *in vitro* or *in vivo* has not previously been evaluated in *B*. *burgdorferi*.

### *B*. *burgdorferi gpsA* complements the growth defect of an *Escherichia coli gpsA* mutant

**Fig 2:**
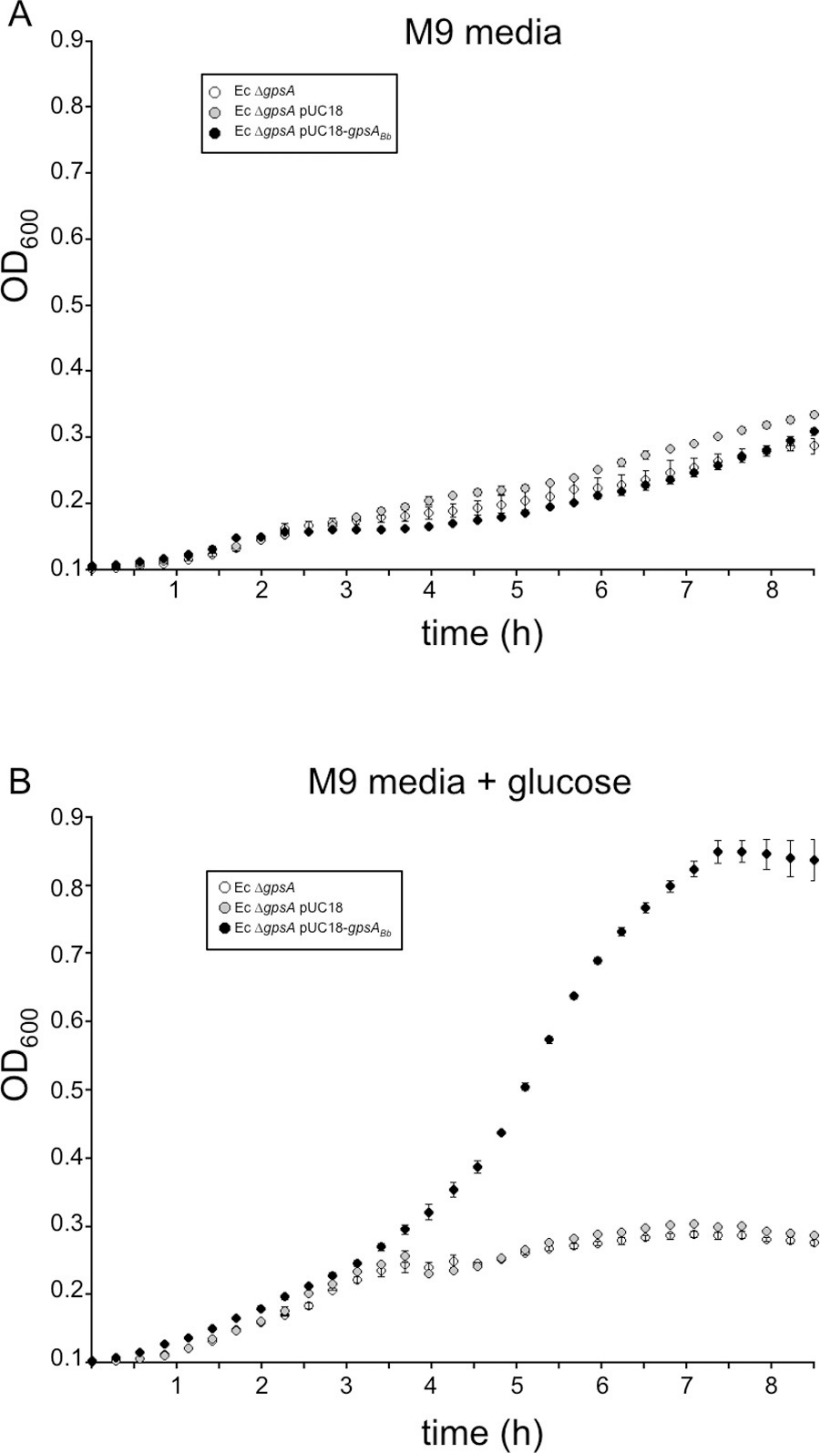
Heterologous complementation of an *E*. *coli gpsA* mutant with *B*. *burgdorferi gpsA* restores growth in glucose. The *E*. *coli gpsA* null mutant, strain BB20-14 (Ec Δ*gpsA*, white circles), *E*. *coli gpsA* null mutant with the inducible pUC18 expression vector (Ec Δ*gpsA* pUC18, gray circles) or *E*. *coli gpsA* null mutant with pUC18 carrying the *B*. *burgdorferi gpsA* gene (Ec Δ*gpsA* pUC18-*gpsA*_Bb_) were grown in M9 minimal media containing 0.1 mM IPTG either without (A) or with 1% glucose (B) at 37°C. Cell density measurements (OD_600_) were taken every 17 min. Data are the average from two separate cultures for each strain and error bars represent SEM; the experiment shown is representative of three independent biological replicates. The Ec Δ*gpsA* pUC18-*gpsA*_Bb_ strain had significantly higher (*p* < 0.05) OD_600_ values compared to the other two strains from 4 h to 8 h of growth, as determined by one-way ANOVA with a Tukey’s *post-hoc* test.

To genetically confirm the predicted function of *B*. *burgdorferi* GpsA, we heterologously complemented the growth phenotype of an *E*. *coli gpsA* mutant. The *B*. *burgdorferi gpsA* (*bb0368*) gene was cloned into the *E*. *coli* (Ec) isopropyl β-d-1-thiogalactopyranoside (ITPG)-inducible expression vector pUC18. The *E*. *coli gpsA* mutant BB20-14 [25], which is a G3P auxotroph and cannot grow on glucose as the sole carbon source, was made competent and transformed with either the empty vector, pUC18, or the vector carrying the *B*. *burgdorferi gpsA* gene, pUC18-*gpsA*_Bb_. The strains Ec Δ*gpsA* (BB20-14), Ec Δ*gpsA*+pUC18 (empty vector) and Ec Δ*gpsA*+pUC18-*gpsA*_Bb_ (expressing *B*. *burgdorferi gpsA*) were inoculated at 5 × 10^7^ cells ml^-1^ and grown in M9 minimal salts media (Fig 2A) or M9 minimal salts media with glucose (Fig 2B). Cultures were grown at 37°C for 9 h with the OD_600_ taken every 17 min. In M9 media lacking a carbon source, all three strains grew at approximately the same rate and failed to grow exponentially or reach high cell density (Fig 2A). When glucose was added as the sole carbon source to the M9 media, only the strain expressing *B*. *burgdorferi gpsA* (Ec Δ*gpsA*+pUC18-*gpsA*_Bb_) had sustained exponential growth and grew to high cell density (>5 × 10^8^ cells ml^-1^) (Fig 2B). These data demonstrate that *B*. *burgdorferi gpsA* can heterologously complement the growth phenotype of an *E*. *coli gpsA* mutant on glucose as a sole carbon source, providing experimental support for the annotated function of *gpsA* in *B*. *burgdorferi*.

### Δ*gpsA*, Δ*glpD* and Δ*gpsA*/Δ*glpD* mutant strains have wild-type growth *in vitro*

**Fig 3:**
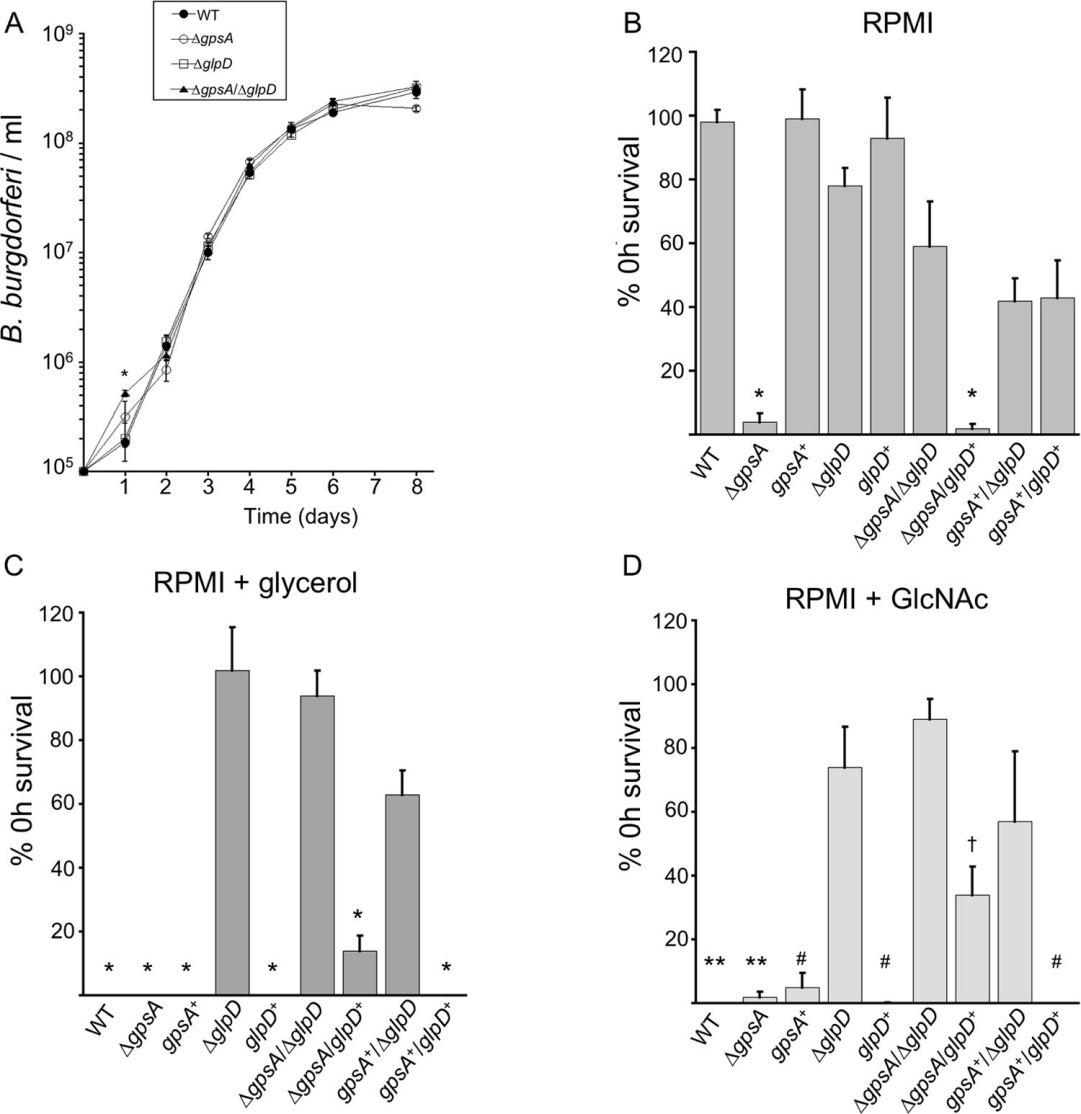
Growth of the *gpsA* and *glpD* mutants and survival under nutrient stress without or with different carbon sources *in vitro*. (A) *B*. *burgdorferi* strains were inoculated at 1 × 10^5^ cells ml^-1^ in BSK + RS and grown at 35°C. Cells were enumerated every 24 h and cell density plotted over eight days. Data are the means from three independent biological replicates and error bars represent SEM. No significant difference in cell density was determined except at day 1 between the wild-type (WT) and **Δ***gpsA*/Δ*glpD* strains: * indicates *p* = 0.046 between the mean cell density of WT and Δ*gpsA*/Δ*glpD* strains at day 1 as determined by one-way ANOVA with a Tukey’s *post-hoc* test. Strains were grown in BSK + RS at 35°C (to a cell density of 5–9 × 10^7^ cells ml^-1^) before shifting to RPMI alone (B), or RPMI containing 0.4% glycerol (C) or 0.4% *N*-acetylglucosamine (GlcNAc) (D) and incubated at 35°C for 24 h. Cultures were plated in semi-solid BSK media and allowed to grow at 35°C in 5% CO_2_ before colony enumeration. Data are presented as the percent survival of each strain before (0 h) shifting to the nutrient stress media. Data are the mean of at least three biological replicates and errors bars represent the SEM. Significance determined by one-way ANOVA with a Tukey’s *post-hoc* test. (* *p* < 0.0001; ** *p* < 0.002; # *p* < 0.01; † *p* = 0.0075).

To investigate the biological role of the metabolic node that interconverts G3P and DHAP regulated by GpsA and GlpD, the genes *gpsA* or *glpD* were mutated alone or together by allelic exchange with antibiotic resistance cassettes to generate Δ*gpsA*, Δ*glpD* and the double mutant Δ*gpsA*/Δ*glpD* strains. All strains were complemented in *cis* to yield the full array of complemented strains: *gpsA* complemented (*gpsA*^+^), *glpD* complemented (*glpD*^+^), *gpsA* complemented in the Δ*gpsA*/Δ*glpD* background (*gpsA*^+^/Δ*glpD*), *glpD* complemented in the Δ*gpsA*/Δ*glpD* background (Δ*gpsA*/*glpD*^+^) and both *gpsA* and *glpD* complemented in the Δ*gpsA*/Δ*glpD* background (*gpsA*^+^/*glpD*^+^) (S1 Fig). Immunoblot analyses of the mutants using antibodies against GlpD and GpsA were used to confirm the deletion and complementation of the respective genes (S1H and S1I Fig). To genetically evaluate the role of *gpsA* and *glpD* during *in vitro* growth, cultures were grown in Barbour-Stoenner-Kelly II media containing 6% rabbit serum (BSK + RS) for eight days and spirochetes were enumerated using a Petroff-Hauser cell counter. No statistical difference in growth was observed between wild-type, Δ*gpsA*, Δ*glpD* or the double mutant Δ*gpsA*/Δ*glpD* strains after day one (Fig 3A). These data suggest that neither *gpsA* nor *glpD* play a significant role during *B*. *burgdorferi* growth in nutrient-rich media *in vitro*.

### GpsA is required for survival during nutrient stress

We hypothesized that GpsA functions in survival during nutrient stress, an *in vitro* condition used to mimic the tick midgut environment between bloodmeals. To test this, *B*. *burgdorferi* strains were grown to 7–9 × 10^7^ cells ml^-1^ in normal growth medium, collected, resuspended and incubated in RPMI medium (which contains 2 mg ml^-1^ glucose) for 24 h, as previously described [18]. Cells were plated in semi-solid BSK, and colonies allowed to grow for approximately two weeks before enumeration. *B*. *burgdorferi* survival is represented as the percentage survival after 24 h of nutrient stress compared to cells plated before nutrient stress (0 h). Δ*gpsA* mutants were almost completely compromised for survival during nutrient stress, while the Δ*glpD* mutant was not significantly affected compared to wild type (Fig 3B). This phenotype is restored in the *gpsA*^+^ and, surprisingly, in the Δ*gpsA*/Δ*glpD* double mutant (Fig 3B). Complementing the double mutant with *glpD*, thus essentially constructing an independent *gpsA* mutant, also resulted in a *B*. *burgdorferi* strain unable to survive nutrient stress, similar to the Δ*gpsA* mutant (Δ*gpsA*/*glpD*^+^ Fig 3B). *gpsA* complementation of the double mutant and complete complementation of the double mutant significantly increased survival compared to Δ*gpsA*, but did not fully restore survival to wild-type levels (*gpsA*^+^/Δ*glpD* and *gpsA*^+^/*glpD*^+^, Fig 3B). These data suggest that *gpsA* plays a crucial role in survival during nutrient stress in culture while *glpD* is dispensable. Furthermore, our results suggest we have identified the first suppressor mutation in *B*. *burgdorferi* as deleting the *glpD* gene in a Δ*gpsA* mutant background restored viability under nutrient stress.

Because the link from glycolysis to G3P metabolism is severed in the Δ*gpsA* mutant, we determined if glycerol could restore survival of the *gpsA* mutant in nutrient stress medium (RPMI + glycerol). Strains were grown and treated as in Fig 3B except that 0.4% glycerol was added to the RPMI medium. Unexpectedly, glycerol in the nutrient stress medium was cytotoxic to wild-type *B*. *burgdorferi* (Fig 3C). In fact, glycerol was toxic to all strains except the Δ*glpD* mutants (Fig 3C), suggesting that metabolism of G3P by GlpD is necessary for the bactericidal activity of glycerol in this restrictive medium. Next, we examined if GlcNAc, a carbohydrate necessary for *in vitro* growth [9,10,26], could rescue the *gpsA* survival defect in RPMI medium. The results with GlcNAc were similar to those with glycerol: survival in GlcNAc was significantly compromised in almost all strains compared to those lacking *glpD* (Fig 3D). The *glpD* complement of the double Δ*gpsA*/Δ*glpD* mutant (Δ*gpsA*/*glpD*^+^) was significantly compromised for survival compared to the Δ*gpsA*/Δ*glpD* mutant (Fig 3D). GlcNAc is not predicted to be metabolized by the action of GlpD, suggesting an unidentified link between GlpD and the metabolism of GlcNAc, possibly involving redox cofactors involved in the GlpD/GpsA oxidoreductase node. Together these results illuminate the importance of the metabolic balance of the intermediates and cofactors involved in the GpsA/GlpD node in *B*. *burgdorferi* adaptation to and survival during changing carbohydrate availability.

### The *gpsA* mutant displays increased round body formation and decreased reductase activity

**Fig 4:**
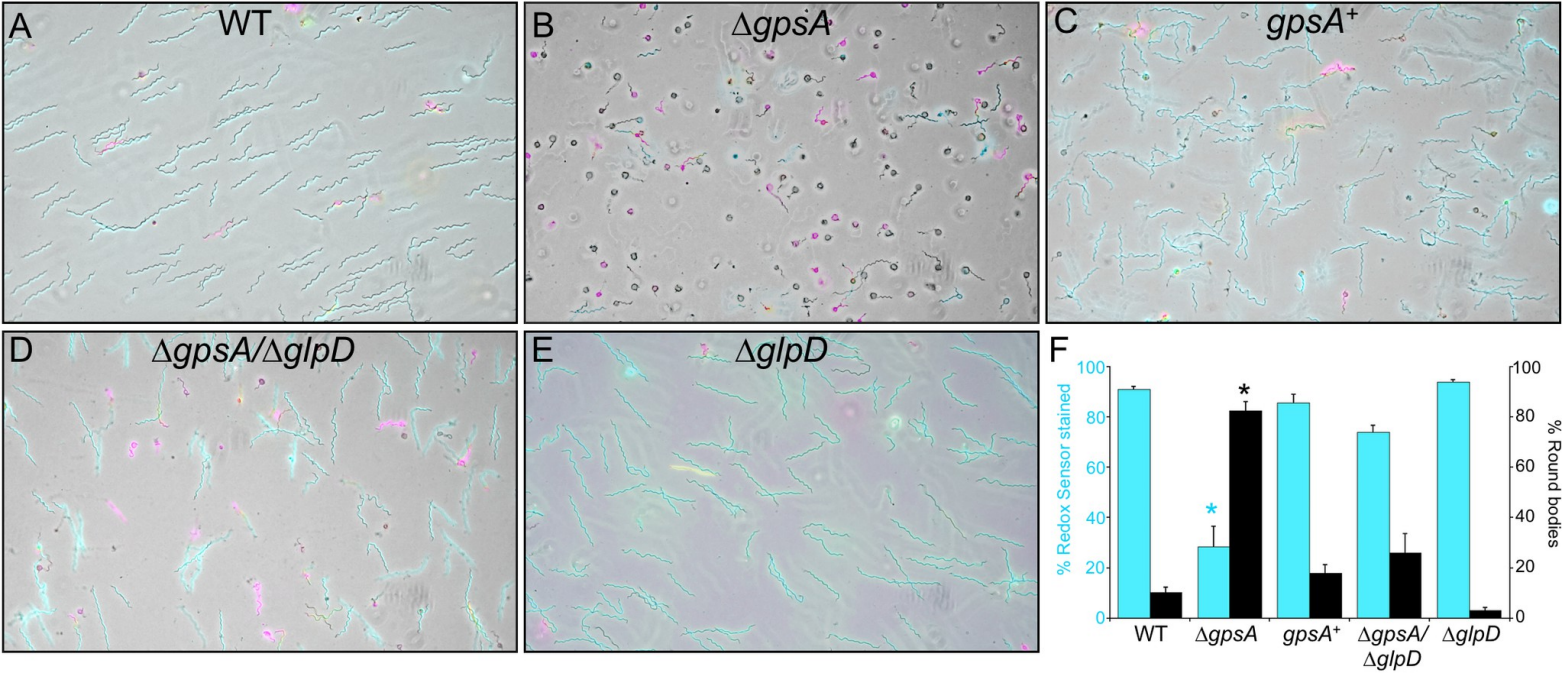
Decreased reductase activity and increased round body formation in the *gpsA* mutant. (A) Wild-type (WT), (B) **Δ***gpsA* mutant, (C) *gpsA*^+^ complement, (D) **Δ***gpsA*/Δ*glpD* double mutant and (E) Δ*glpD* mutant strains were grown in BSK + RS at 35°C (to a cell density of 5–9 × 10^7^ cells ml^-1^) before shifting to RPMI for 16 h at 35°C. Bacterial reductase activity was detected by staining with RedoxSensor Green and membrane integrity assessed by staining with propidium iodide (PI). Live cells were imaged by fluorescence microscopy for RedoxSensor Green (cyan) and PI (magenta) and overlaid with white light images. (F) The percentage of cells stained with RedoxSensor Green and the percentage of cells in round body (RB) form was quantified. Data are the mean of three biological replicates and error bars represent the SEM. Asterisks signify a significant difference (*p* ≤ 0.0001) between the mean percent of RedoxSensor-stained Δ*gpsA* cells compared to all other strains and the mean percent of RBs in the Δ*gpsA* strain compared to all other strains as determined by one-way ANOVA with a Tukey’s *post-hoc* test.

To assess the redox potential of the G3PDH mutants, overall reductase activity in individual cells was monitored by microscopy using a fluorescent reporter. Wild-type and mutant strains were grown to ~7–9 × 10^7^ cells ml^-1^, collected and incubated in RPMI medium (nutrient stress) for 16 h. Cultures were then incubated with Bac*Light* RedoxSensor Green and propidium iodide for 10 min before wet-mounting live cells to be imaged by fluorescence microscopy. RedoxSensor Green stain fluoresces (shown as cyan in Fig 4) when reduced, indicating intracellular reductase activity and cell viability. Almost all (90%) wild-type cells in nutrient stress media for 16 h show reductase activity/viability while only about 30% of Δ*gpsA* cells fluoresce (cyan staining, Fig 4A, 4B, and 4F). Strikingly, Δ*gpsA* cells undergo a dramatic morphological change from flat-wave to a condensed spherical form called round bodies (RBs) during nutrient stress that is rarely seen in wild-type *B*. *burgdorferi* (Fig 4A, 4B, and 4F). The physiological role of RBs remains unknown but the transition is triggered by environmental stress and may be related to persistence, as this form has been observed *in vivo* in ticks [18,27–29]. Both the decrease in reductase activity and the increase in RB formation in Δ*gpsA* mutant cells are restored not only in the *gpsA*^+^ strain but also in the Δ*gpsA*/Δ*glpD* double mutant (Figs 4C, 4D, and 4F), which is further evidence of *glpD* functioning as a suppressor mutation of the pleiotropic *gpsA* phenotypes. Deletion of the *glpD* gene alone affected neither reductase activity nor morphology during nutrient stress (Fig 4E and 4F). These data further support the findings that GpsA is important for cell survival under nutrient stress, a result likely reflected in reduced reductase activity. Additionally, the dramatic increase in RB formation of the Δ*gpsA* mutant is evidence that GpsA is a key modulator of morphological changes during nutrient stress.

A recent study has implicated GpsA in resistance of *Streptococcus pneumoniae* to oxidative stress [30]. To examine if *gpsA* also protects *B*. *burgdorferi* from oxidative stress, we measured the viability of wild-type, Δ*gpsA* and *gpsA*^+^ strains following exposure to H_2_O_2_ and found no significant differences between the strains, at least under the conditions tested (S2 Fig).

### GpsA and GlpD influence the *B*. *burgdorferi* metabolome

**Fig 5:**
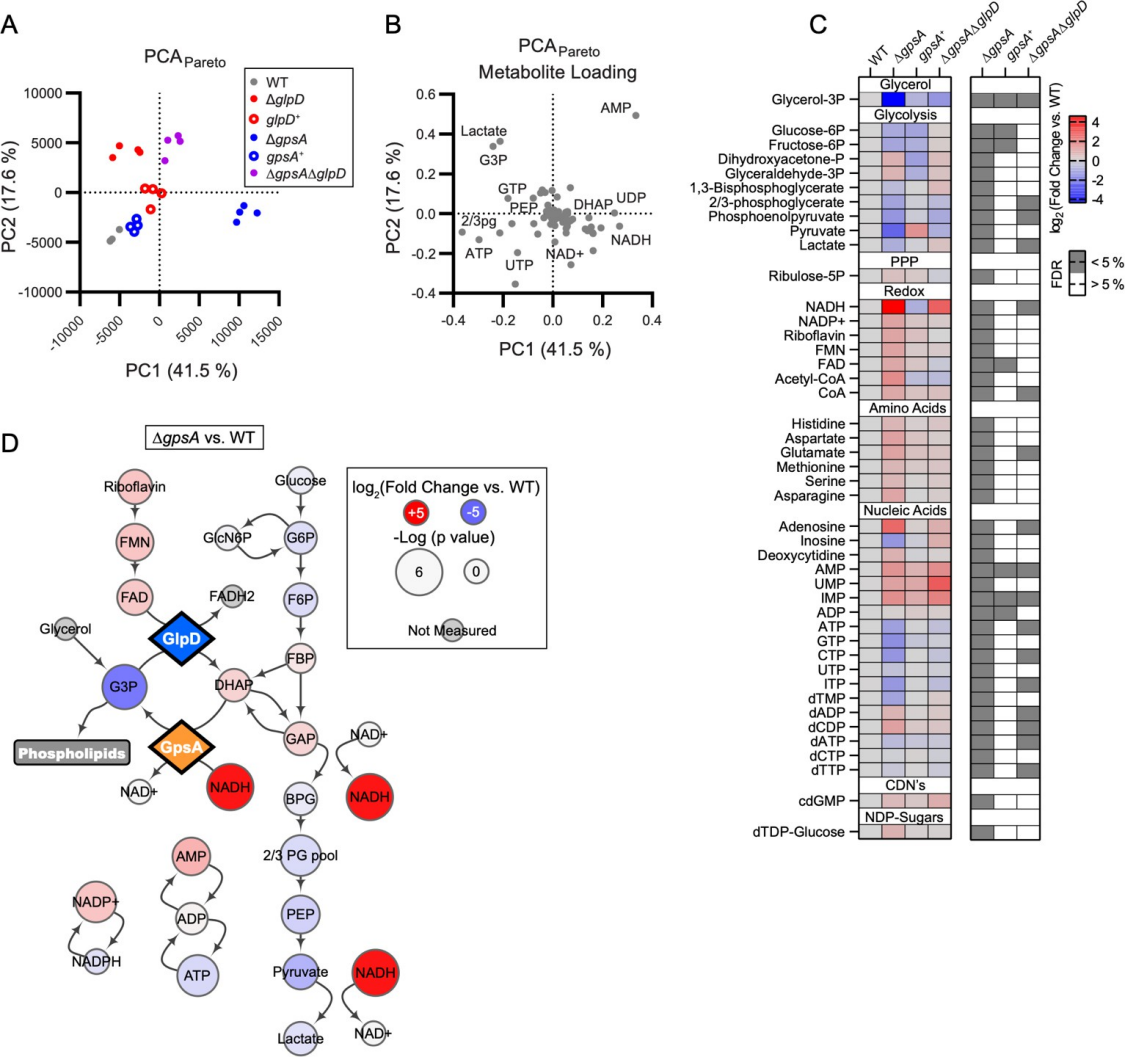
GpsA regulates central metabolism and redox balance. (A) Polar metabolomics of bacterial strains subjected to principal component (PC) analysis with pareto-scaling. (B) The corresponding metabolite loading distribution for the analysis displayed in (A) for principal components 1 and 2. (C) Metabolites that significantly vary between Δ*gpsA* and wild type (WT) with a false discovery rate (FDR) less than 5%. Values in the heatmap at left are displayed as the log_2_(fold change mutant versus WT) and values in the accompanying heatmap at right indicate whether that metabolite passes a 5% FDR filter for the indicated comparison as assessed by a Benjamini-Hochberg correction. (D) Metabolic map of the changes in glycolysis and the glycerol shunt that occur with the loss of GpsA. All measured metabolites in the included pathways are displayed. The log_2_(fold change Δ*gpsA* versus WT) is displayed as color of the node and the -log(*p*-value) is displayed as the size of the node. Enzymes in the glycerol arm of metabolism are displayed as diamonds. Data are from four independent biological replicates.

To better understand how GpsA and GlpD affect the global physiology of *B*. *burgdorferi*, we performed semi-targeted metabolomics by liquid chromatography-tandem mass spectroscopy on the Δ*gpsA* and Δ*glpD* mutants in culture. Strains were grown in BSK + RS at 35°C to a density of ~3 × 10^7^ cells ml^-1^ before processing and analysis. Comparing the mutant metabolomes to wild type by unbiased principal component analysis, separate axes were readily observed that defined the metabolic effects of Δ*gpsA* versus the metabolic effects of Δ*glpD*. Complements of both mutants resulted in a return toward wild type and the Δ*gpsA*/Δ*glpD* double mutant comigrated with Δ*glpD* in agreement with the increased survival phenotype of the Δ*gpsA*/Δ*glpD* double mutant (Fig 5A). The first principal component, which defines the separation of Δ*gpsA* from wild type, is heavily loaded by the opposing behavior of ATP and AMP, positively loaded for NADH, and negatively loaded with G3P and three-carbon glycolytic intermediates (Fig 5B). By contrast, Δ*glpD* separation along principal component two is positively loaded for G3P and negatively loaded for NADH in support of an opposing reaction directionality between GpsA and GlpD. Interestingly, both primary principal components are positively loaded for AMP and negatively loaded for ATP suggesting that proper functioning of the G3P arm of *B*. *burgdorferi* metabolism is essential for energy metabolism (Fig 5B).

In further support of the anticipated enzymatic function of GpsA, univariate analysis of the metabolite datasets showed that the largest metabolic changes in the Δ*gpsA* mutant compared to wild type were localized to the putative redox cofactor (NADH, 23.7-fold increase) and the anticipated product (G3P, 20.5-fold decrease) of GpsA (Fig 5C). Despite its predicted role as a substrate of GpsA, DHAP levels were only 1.7-fold higher in the Δ*gpsA* mutant. This smaller effect compared to G3P and NADH is likely due to triose phosphate isomerase converting DHAP to glyceraldehyde-3-phosphate (GAP) for glycolysis (Fig 5D). With the exceptions of DHAP, GAP, and fructose-1,6-bisphosphate, glycolytic intermediates, particularly pyruvate (6.6-fold decrease), were decreased in the Δ*gpsA* mutant. Concomitantly, ATP levels decreased (2.1-fold) and AMP levels increased (2.6-fold). These changes in glycolysis likely drive the decreased energy levels in the cell and may account for the susceptibility of the Δ*gpsA* mutant strain to nutrient stress (Fig 3). The complete list of metabolite levels in all the mutants is included (S2 Table).

The dramatic 23.7-fold increase in NADH levels in the *gpsA* mutant compared to wild type suggests that GpsA is a dominant regulator of NADH levels in *B*. *burgdorferi*. The dysregulation of glycolysis may be a consequence of these elevated levels of NADH, which would inhibit glyceraldehyde-3-phosphate dehydrogenase (GAPDH) and thus glycolysis in the Δ*gpsA* mutant [31] (Fig 5D). The changes in metabolite levels in the Δ*gpsA* mutant were largely restored to wild-type levels in the *gpsA*^+^, but energy stress was still apparent in elevated AMP levels (Fig 5C). Metabolite levels in the Δ*gpsA*/Δ*glpD* double mutant shifted toward wild-type levels, although not as completely as in the *gpsA*^+^. Univariate analysis further supported the action of GlpD as metabolically opposed to GpsA. While the metabolic phenotype in the Δ*glpD* mutant was of a smaller magnitude compared to the Δ*gpsA* mutant, DHAP levels in the Δ*glpD* mutant were 4.9-fold lower and G3P levels 4.2-fold higher than in the wild type (S3A Fig), supporting the predicted GlpD function. NADH levels were lower in the Δ*glpD* mutant, suggesting that either NADH is the reduced cofactor as G3P is oxidized to DHAP or that the effects of *glpD* deletion on GAP limit the recovery of NADH by GAPDH (S3B Fig). The effects on glycolysis in the Δ*glpD* mutant were localized to DHAP, GAP, phosphoenolpyruvate, and pyruvate (S3A Fig). Complementation of the Δ*glpD* mutant overcorrected the elevated levels of G3P and somewhat restored levels of NADH associated with *glpD* deletion suggesting the reintroduction of active enzyme (S3A Fig). However, dysregulation of glycolysis and energy metabolism was not restored between the Δ*glpD* mutant and the *glpD*^+^, indicating that there was only partial restoration of the wild-type phenotype even though *glpD* was complemented in *cis* with the native *glp* promoter.

### GpsA regulates the NADH/NAD^+^ ratio

**Fig 6:**
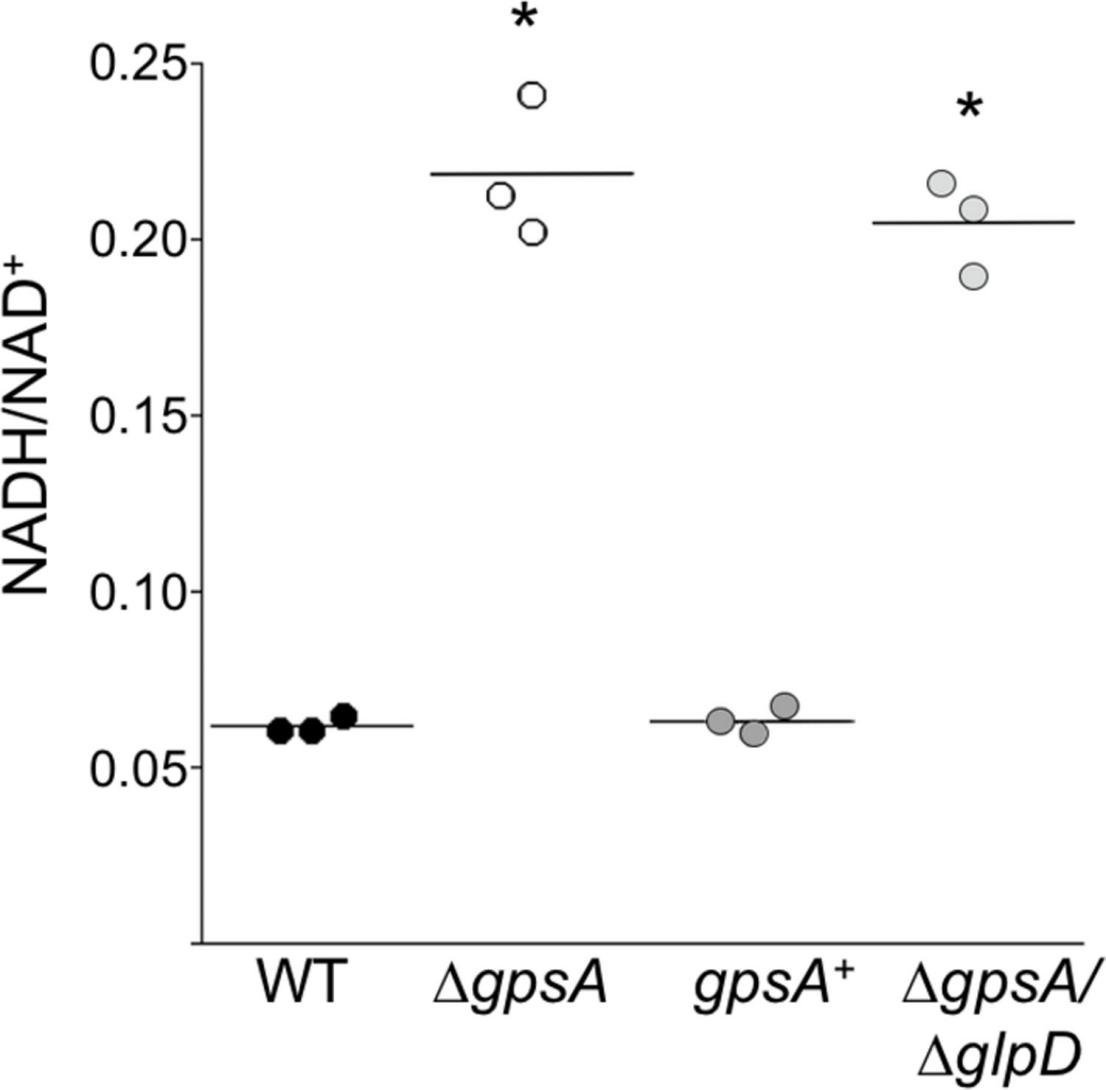
GpsA regulates NADH/NAD^+^ levels. Wild-type (WT, black circles), Δ*gpsA* mutant (Δ*gpsA*, white circles), *gpsA* complemented (*gpsA*^+^, dark gray circles) and Δ*gpsA*/Δ*glpD* double mutant (light gray circles) strains were grown in BSK + RS at 35°C to ~5 × 10^7^ cells ml^-1^ and NAD^+^ and NADH levels were measured with the NAD/NADH-Glo Assay. Each point represents a single biological replicate and bars represent the means. Asterisks represent a significant difference (*p* < 0.0001) in the means of NADH/NAD^+^ molar ratios of both the Δ*gpsA* mutant and the Δ*gpsA*/Δ*glpD* double mutant compared to those of both the wild type and the *gpsA*^+^ determined by one-way ANOVA with a Tukey’s *post-hoc* test.

To confirm and quantify the GpsA-mediated regulation of nicotinamide cofactor levels observed in the metabolomics analysis, the NADH/NAD^+^ molar ratios in the Δ*gpsA* mutant were measured via an *in vitro* luminescence assay. Strains were grown and samples prepared as described for the metabolomics studies before measuring the NADH and NAD^+^ levels. The NADH/NAD^+^ molar ratio was approximately fourfold higher in the Δ*gpsA* mutant strain than in wild-type cells (Fig 6). This difference was fully restored in the *gpsA*^+^, but, curiously, not in the double Δ*gpsA*/Δ*glpD* mutant strain (Fig 6). These data support the finding that *gpsA* highly regulates NADH levels in *B*. *burgdorferi* (Fig 5).

### GpsA and GlpD levels are independent of each other

**Fig 7:**
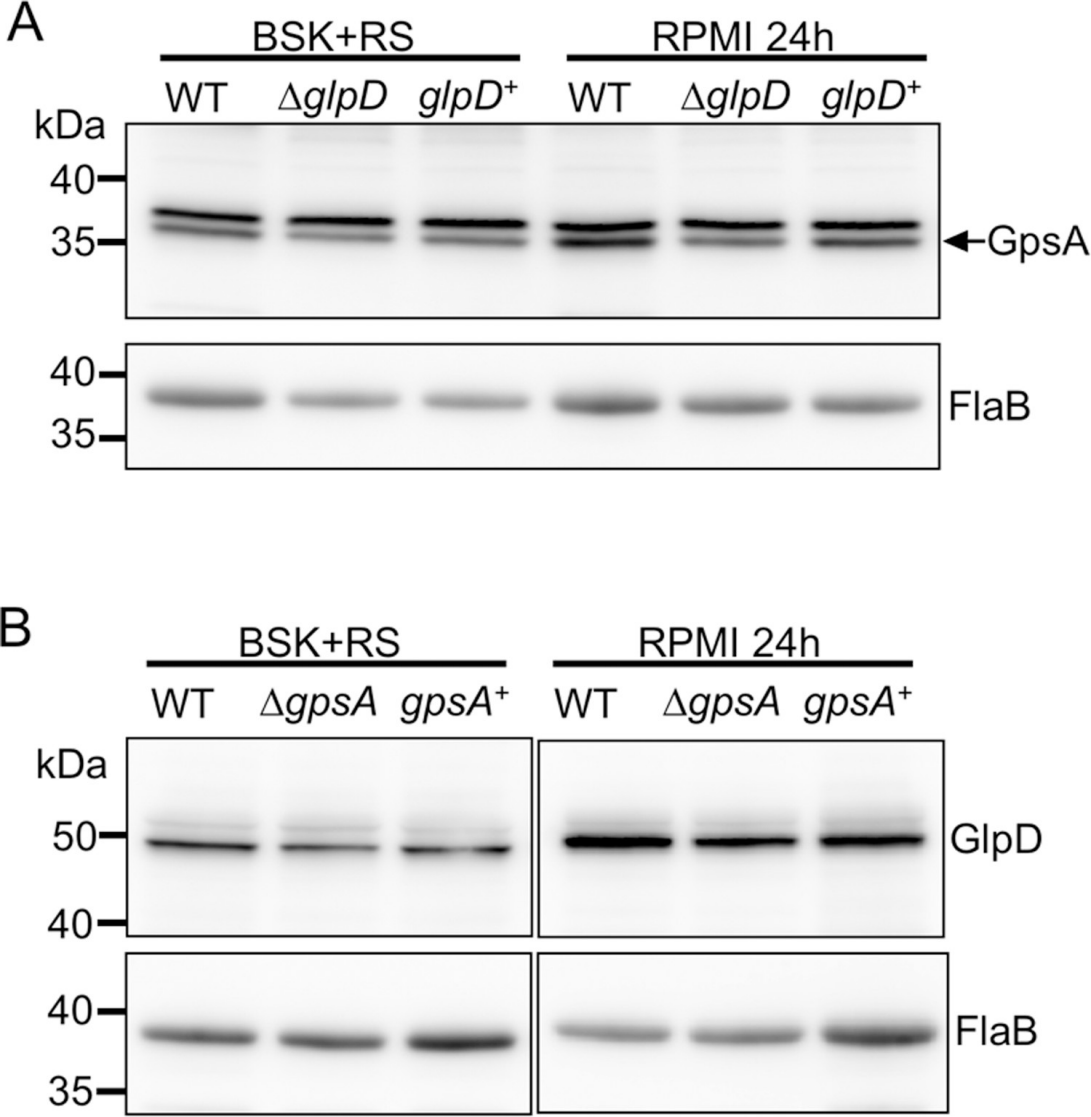
GpsA and GlpD levels are independent. (A) Wild-type (WT), Δ*glpD* mutant (Δ*glpD*) and *glpD* complemented (*glpD*^+^) strains were grown in BSK + RS at 35°C or shifted to nutrient stress medium (RPMI) for 24 h before total cell lysates were collected. Samples were separated by SDS-PAGE and analyzed by immunoblot with antibodies against GpsA or FlaB (as a control). GpsA is the lower band of the doublet (see S1H Fig). (B) WT, Δ*gpsA* mutant (Δ*gpsA*) and *gpsA* complemented (*gpsA*^+^) strains were grown and analyzed as in (A) except antibodies against GlpD were used for the immunoblots in the upper panels. Three independent experiments were done and representative data are shown.

Because GpsA and GlpD catalyze the interconversion of G3P and DHAP, *B*. *burgdorferi* may respond to the mutation of one enzyme by altering the levels of the other G3PDH to compensate. To examine this possibility, levels of GpsA protein were examined in the Δ*glpD* mutant and levels of GlpD protein were examined in the Δ*gpsA* mutant by immunoblot analyses. Cell lysates analyzed from wild-type, Δ*glpD* mutant and *glpD*^+^ strains grown in normal growth medium (BSK + RS) at 35°C or incubated in nutrient stress medium (RPMI) showed no appreciable difference in GpsA protein levels (Fig 7A). FlaB was used as a loading control (Fig 7A and 7B). Similarly, GlpD levels did not change in the Δ*gpsA* mutant compared to the wild type or the *gpsA*^+^ under similar conditions (Fig 7B). Thus, GpsA and GlpD protein levels are independent of each other, at least during *in vitro* culture.

### GpsA is required for murine infectivity

Because GpsA and GlpD dramatically affect *B*. *burgdorferi* metabolism, particularly NADH levels, we examined the role of this oxidoreductase cycle *in vivo* using the mouse-tick model of Lyme disease. To genetically assay the function of GpsA and GlpD in murine infection, 10^4^ cells of wild-type, Δ*gpsA*, *gpsA*^+^, Δ*glpD*, *glpD*^+^ and Δ*gpsA*/Δ*glpD* strains were intradermally needle-inoculated in C3H-HeJ mice. Infection and dissemination were assessed via ear biopsies three weeks post-inoculation and tissues were cultured for *B*. *burgdorferi*. None of the mice injected with the Δ*gpsA* mutant were infected (Table 1).

**Table 1:** Infectivity by intradermal needle inoculation.

# cells injected	Strain	3 weeks		5 weeks	
		Ear	Ear	Ankle	Bladder
**1 × 10^4^**	WT	3/4	3/4	3/4	3/4
**1 × 10^4^**	*ΔgpsA*	0/4	0/4	0/4	0/4
**1 × 10^4^**	*gpsA* ^+^	4/4	4/4	4/4	4/4
**1 × 10^4^**	*ΔglpD*	4/4	4/4	4/4	4/4
**1 × 10^4^**	*glpD* ^+^	3/4	3/4	3/4	3/4
**1 × 10^4^**	*ΔgpsA*/*ΔglpD*	0/4	0/4	0/4	0/4
**1 × 10^5^**	*ΔgpsA*	0/4	0/4	0/4	0/4
**1 × 10^6^**	*ΔgpsA*	0/4	0/4	0/4	0/4
**1 × 10^5^**	*ΔgpsA*/*ΔglpD*	0/4	3/4	4/4	3/4
**1 × 10^6^**	*ΔgpsA*/*ΔglpD*	0/4	4/4	4/4	2/4

Infectivity was restored in the *gpsA*^+^ but not in the Δ*gpsA*/Δ*glpD* double mutant. Ear, ankle and bladder tissues were collected from the same mice five weeks post inoculation and cultured for *B*. *burgdorferi* to follow infectivity and dissemination. The results at five weeks were consistent with those at three weeks: the Δ*gpsA* mutant and Δ*gpsA*/Δ*glpD* double mutant were still unable to establish infection (Table 1). Because GpsA is potentially involved in providing G3P for lipoprotein biosynthesis, we examined the levels of two lipoproteins, outer surface proteins A and C (OspA and OspC), in the Δ*gpsA* and Δ*gpsA*/Δ*glpD* mutants. In strains grown *in vitro*, under the same conditions used for mouse infectivity studies, there was no appreciable difference in the levels of OspC or OspA (S4 Fig). The absence of *glpD* did not affect murine infectivity by needle inoculation, results agreeing with previous studies [12,13].

To determine if the infectivity defect of the Δ*gpsA* and Δ*gpsA*/Δ*glpD* mutants could be overcome by increasing the challenge dose, mice were inoculated with 10^5^ or 10^6^ cells of each strain, and infectivity and dissemination monitored as described above at three and five weeks after inoculation. The Δ*gpsA* mutant was noninfectious even with a dose of 10^6^ cells (Table 1). At three weeks post challenge, *B*. *burgdorferi* could not be isolated from any mice injected with either 10^5^ or 10^6^ of Δ*gpsA*/Δ*glpD* double mutants (Table 1). Surprisingly, five weeks after challenge with the higher doses of the Δ*gpsA*/Δ*glpD* double mutant, 20/24 tissues were positive for *B*. *burgdorferi* re-isolation, indicating that deletion of *glpD* suppresses the non-infectious phenotype of the Δ*gpsA* mutant, thus partially restoring infectivity, albeit with delayed kinetics. These data provide additional *in vivo* evidence that *glpD* functions as a suppressor mutation of the non-infectious phenotype of the Δ*gpsA* mutant.

### GpsA is crucial for persistence in the tick and transmission to mice

**Fig 8:**
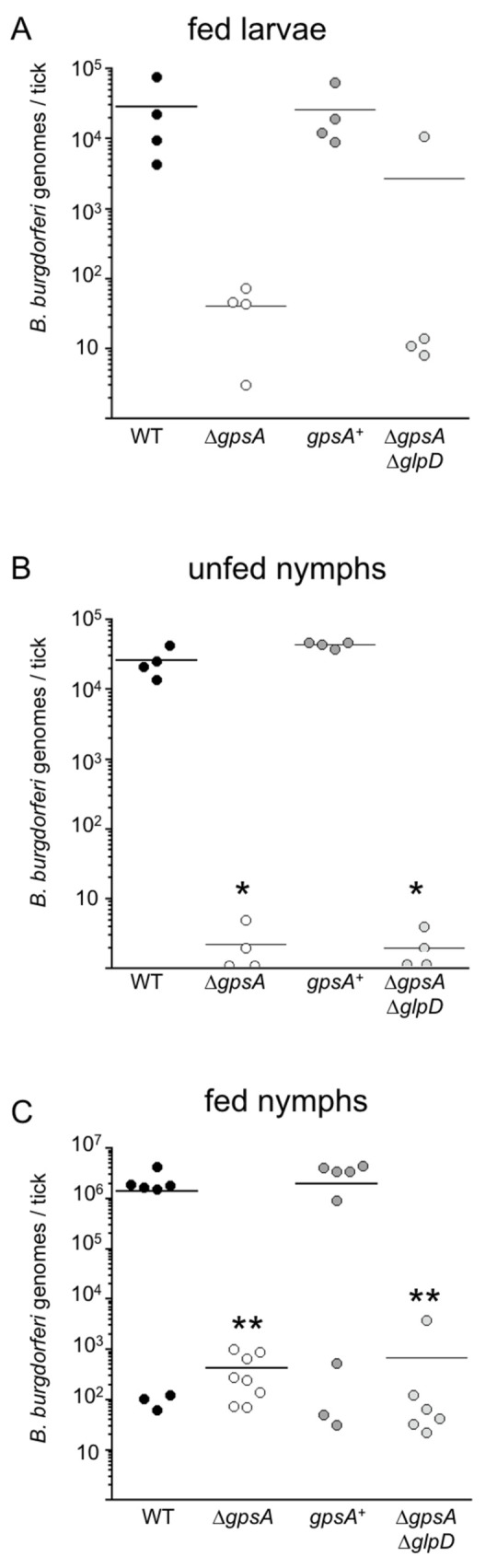
Persistence of the *gpsA* and *glpD* mutants in ticks. *B*. *burgdorferi* strains were introduced to larval ticks by immersion infection and fed on naïve mice. Acquisition and persistence of wild type (WT, black circles), Δ*gpsA* mutant (white circles), *gpsA* complement (*gpsA*^+^) (dark gray circles) and Δ*gpsA*/Δ*glpD* double mutant (light gray circles) was assessed in ticks one week after larvae fed to repletion (A), following the molt to nymphs, nine weeks later (B) and one week after nymphs fed to repletion on naïve mice (C). DNA from infected ticks was isolated at each stage and *B*. *burgdorferi* load in ticks measured by TaqMan qPCR using primers/probe to *flaB*. Bars represent the mean of data points from a single experiment. The means of both the Δ*gpsA* and Δ*gpsA*/Δ*glpD* mutants were significantly different (* *p* < 0.0007) from both the wild type and the *gpsA*^+^ determined by one-way ANOVA with a Tukey’s *post-hoc* test in (B). The mean of the *gpsA*^+^ was significantly different (** *p* ≤ 0.03) from both Δ*gpsA* and Δ*gpsA*/Δ*glpD* mutants as determined by one-way ANOVA with a Tukey’s *post-hoc* test in (C).

*gpsA* is essential for survival during nutrient stress during *in vitro* cultivation (Fig 3B, 3C, and 3D); therefore, we hypothesized that *gpsA* functions *in vivo* during persistence in the tick, when the spirochetes experience nutrient limitation after the tick absorbs the blood meal. To test this hypothesis, we artificially infected naïve larvae with wild-type, Δ*gpsA*, *gpsA*^+^ and Δ*gpsA*/Δ*glpD* strains as previously described [32]. *B*. *burgdorferi* loads were measured in ticks through maturation and quantified by qPCR using primers to the *B*. *burgdorferi flaB* gene on DNA isolated from infected ticks. The Δ*gpsA* mutant was unable to establish a robust colonization in larvae compared to wild type and this deficiency was restored in the *gpsA*^+^ (Fig 8A). The Δ*gpsA*/Δ*glpD* double mutant was also compromised for colonization of larvae, indicating that this Δ*gpsA* mutant phenotype was not suppressed by the deletion of *glpD* (Fig 8A). Persistence through the molt from fed larvae to flat nymphs continued to be compromised in both the Δ*gpsA* mutant and the Δ*gpsA*/Δ*glpD* double mutant compared to wild type and the *gpsA*^+^, as *B*. *burgdorferi* loads fell to an average of less than 10 per tick (Fig 8B). Following feeding of infected nymphs on naïve mice, *B*. *burgdorferi* levels in Δ*gpsA* and Δ*gpsA*/Δ*glpD*-infected ticks increased but remained greater than three orders of magnitude below those found in wild type and *gpsA*^+^-infected fed nymphs (Fig 8C). These *in vivo* data demonstrate a crucial, although not essential, role for *gpsA* to colonize and persist in ticks in the animal model of Lyme disease.

Transmission by tick bite is the natural route of *B*. *burgdorferi* infection of vertebrates. To examine the role of *gpsA* in transmission of *B*. *burgdorferi* from nymphs to naïve mice, three nymphs per mouse were allowed to feed until repletion. Murine infections were monitored three and five weeks post-feeding by culturing collected murine tissues as described above. The Δ*gpsA* mutant was not transmitted to mice (0/3 mice infected) and this defect was restored in the *gpsA*^+^ strain (2/3 mice infected) (Table 2). The Δ*gpsA*/Δ*glpD* double mutant was also compromised for transmission (0/3 mice infected) (Table 2), suggesting that *glpD* does not suppress the *gpsA* mutation in tick transmission.

**Table 2:** Infectivity by nymph bite.

strain	3 weeks		5 weeks	
	Ear	Ear	Ankle	Bladder
**WT**	3/3	3/3	3/3	3/3
***ΔgpsA***	0/3	0/3	0/3	0/3
***gpsA* ^+^**	2/3	2/3	2/3	2/3
***ΔgpsA*/*ΔglpD***	0/3	0/3	0/3	0/3

## Discussion

We have provided experimental evidence that the *B*. *burgdorferi gpsA* encodes a G3PDH, as predicted [5], by heterologously complementing an *E*. *coli gpsA* mutant (Fig 2). Our metabolomics analyses suggest that GpsA converts DHAP to G3P, as the Δ*gpsA* mutant showed reduced levels of G3P and increased levels of DHAP and NADH (Fig 5), providing additional evidence that *gpsA* encodes a G3PDH (or, based on the directionality of the reaction, a DHAP reductase). We also identified NADH as the likely cofactor of the redox reaction. Previous studies have shown that GlpD plays a role in growth on glycerol, but the substrates and products of GlpD have not been experimentally examined [12,13,15]. Here, our metabolomics analyses of the *glpD* mutant demonstrate a significant increase of G3P and decrease of DHAP levels compared to wild type, experimental evidence suggesting GlpD oxidizes G3P to DHAP. The lower NADH levels seen in the Δ*glpD* strain suggest that NAD^+^ serves as the cofactor for oxidation of G3P by GlpD. An alternative possibility is that NAD^+^ is reduced downstream by a different cofactor, such as FADH_2_, used by GlpD.

### Metabolic virulence

The intersection of metabolism and virulence in microbial pathogens is gaining attention in the quest to define the molecular mechanisms responsible for causing disease [33]. In particular, bacterial glycerol metabolism and the associated physiological landscape can influence disease pathogenesis [34]. In the limited metabolic capacity of *B*. *burgdorferi*, glycerol metabolism plays an important role in the ability of the spirochete to traverse its enzootic cycle and, thus, the glycerol utilization genes are subject to complex regulation [5,7,8,16]. In this work we have presented evidence identifying a physiological linchpin connecting glycerol metabolism/lipid biosynthesis and glycolysis/energy generation that regulates stress survival *in vitro* and is crucial *in vivo* for both murine infectivity and persistence in the tick vector. This oxidoreductive node consists of two opposed G3PDHs, GpsA and GlpD, which catalyze the interconversion of G3P and DHAP, and likely communicates closely with the adjacent GAPDH. We found that *gpsA* is essential for disseminated infectivity in mice, by needle inoculation of up to 10^6^ cells (Table 1), and is crucial for persistence in ticks (Fig 8). Δ*gpsA* mutants and Δ*gpsA*/Δ*glpD* double mutants were also compromised for transmission by nymphs, but the tick transmission phenotype is likely due, at least in part, to reduced spirochete loads in the tick. The infectivity phenotype may result from the inability of these mutant strains to establish infection at the inoculation (or tick bite) site or may reflect a defect in dissemination or persistence, but uncovering these details awaits further investigation. Additionally, a more detailed time course of Δ*gpsA* mutant and Δ*gpsA*/Δ*glpD* double mutant replication during tick feeding may shed light on how these strains are unable to complete the vector-host lifecycle in the laboratory model of Lyme disease.

Recent studies have suggested that *gpsA* may be a virulence factor in other pathogens. Green et al., 2021 [30] found using a forward genetic screen that GpsA is important for *S*. *pneumoniae* to establish nasopharyngeal colonization and plays a role in resistance to oxidative stress. A G3PDH mutant strain of the plant pathogen *Acidovorax citrulli* showed reduced virulence and increased resistance to antibiotics [35]. Additionally, a genome-wide mutagenesis screen identified a *gpsA* mutant in *Bartonella* that was avirulent, but the transposon insertion is upstream of the *gpsA* gene, complicating interpretations of the results [36]. Thus, GpsA activity, converting DHAP to G3P using a redox cofactor such as NADH, while not important for viability and growth under optimized culture conditions, represents a metabolic virulence factor important during the challenges of establishing host infection and resistance to immune defenses.

### GlpD as a suppressor mutant

We have identified the *glpD* mutant as a suppressor of several *gpsA* mutant phenotypes, including murine infectivity by needle inoculation at high doses, restoration of viability under nutrient stress, and morphological changes. Much of the metabolic imbalance observed in our metabolomic studies of the Δ*gpsA* mutant were restored in the Δ*gpsA*/Δ*glpD* double mutant, particularly the dramatic increase in NADH and decrease in G3P levels. Alleviating this imbalance by the absence of GlpD activity, which results in a decrease of NADH levels and an increase in G3P levels, may explain the suppression of the defects in the Δ*gpsA* mutant. There is precedent for the interdependence of *gpsA* and *glpD* in *E*. *coli*. The *gpsA* gene was thought to be essential in *E*. *coli* as mutants were not generated in a genome-wide, single gene deletion library known as the Keio collection [37]. However, deletion of the *gpsA* gene previously had been accomplished in a *glpD* mutant background in *E*. *coli* [38], supporting our assertion that *glpD* suppresses aspects of the pleiotropic *gpsA* mutant phenotype. Together, these findings suggest this node may be a common stress point, although bacteria with a more diverse metabolic repertoire may be better able to adjust for the metabolic imbalances created by genetic perturbation of either of these G3PDHs.

The *glpD* mutant suppressed some, but not all, of the defects of the *gpsA* mutant in the animal model of Lyme disease: infectivity by needle inoculation (10^5^ and 10^6^ cells) was restored, but persistence in ticks and transmission from nymphs to mice were not. These results suggest that certain requirements and/or signals in ticks cannot be overcome by complete disruption of the GpsA/GlpD node and likely require efficient carbon flow between glycolysis and lipid/lipoprotein biosynthesis. Even though levels of the outer membrane lipoproteins OspC and OspA were not affected *in vitro* in the Δ*gpsA* and Δ*gpsA*/Δ*glpD* mutants, there may be differences in lipoprotein production *in vivo* that contribute to the defect in persistence in the tick.

### The GpsA and GlpD metabolomes

Although the host metabolome in response to Lyme disease has been analyzed [39,40], here we report the first broad metabolomic datasets of *B*. *burgdorferi*, consisting of 129 metabolites. Our results identified GpsA as a dominant regulator of NADH levels and redox potential in the spirochete. The 23-fold increase in NADH in the Δ*gpsA* mutant compared to wild type was partially dependent on GlpD activity as levels were largely restored in the Δ*gpsA*/Δ*glpD* double mutant (5.5-fold increase over wild type; Fig 5C). The redox state of the cell, reflected at least in part by the NADH/NAD^+^ ratio, is intimately entwined with glycolysis and secondary carbon metabolism [6,7]. Therefore, changes in the levels of glycolytic intermediates seen in the Δ*gpsA* mutant were likely a secondary effect of extremely high levels of NADH, a cofactor known to inhibit GAPDH activity [31]. This NADH-mediated inhibition could dramatically affect carbon flow in glycolysis. In fact, we found evidence to support this conclusion as levels of the two metabolites upstream of GAPDH, fructose-1,6-bisphosphate and GAP, were elevated and levels of all metabolites downstream were decreased. Again, in the Δ*gpsA*/Δ*glpD* double mutant where levels of NADH were closer to those in wild-type cells, the levels of all intermediates in glycolysis trended back toward wild-type levels, with the majority no longer significantly different. The same trend was observed in energy metabolism as represented by the levels of ATP, which could explain the differing viability *in vitro* under nutrient stress between the Δ*gpsA* mutant and the Δ*gpsA*/Δ*glpD* double mutant (Fig 3B, 3C, and 3D).

The *B*. *burgdorferi* genome lacks the genes encoding the enzymes required for *de novo* NAD^+^ synthesis, but contains homologs of enzymes that convert nicotinamide to NAD^+^ (*pncA*, *pncB*, *nadD* and *nadE*), although a nicotinamide transporter has not yet been identified [5,6]. In the absence of an electron transport chain and reductive biosynthetic processes such as fatty acid synthesis, many routes of NADH oxidation are absent in *B*. *burgdorferi* [7]. The ability of *B*. *burgdorferi* to recycle NADH is further limited by the absence of genes encoding pyruvate dehydrogenase, lactate oxidase and pyruvate formate lyase. NAD^+^ can be recovered by lactate dehydrogenase converting pyruvate, formed in glycolysis, to lactate, which is then excreted from the cell through lactate permease [5]. The reverse reaction of converting lactate to pyruvate would regenerate NADH from NAD^+^. The constraints placed on the NAD^+^/NADH balance in *B*. *burgdorferi* likely result in increased sensitivity to disruption of this ratio and a greater need to recycle both the oxidized and reduced cofactors, particularly in glycolysis where NAD^+^ is used as an electron acceptor. GpsA represents one of the few enzymes identified in *B*. *burgdorferi* able to recycle NADH to NAD^+^, while at the same time supplying G3P, but at a cost to energy production from glycolysis.

NADH-dependent oxidative stress enzymes, such as coenzyme A disulfide reductase (CoADR) and superoxide dismutase (SodA) also can affect the redox state of the cell. CoADR is involved in reducing the disulfide form of coenzyme A occurring in an oxidative environment, using exclusively NADH [41]. The response of CoA-metabolites to *gpsA* deletion suggests that the glycerol oxidoreductase node and the CoADR systems are in metabolic communication, likely via NADH (Fig 5C). CoADR protects *B*. *burgdorferi* from lipid peroxidation, is essential for host infectivity and plays a role in survival in fed nymphs [41,42]. SodA in *B*. *burgdorferi* is required for murine infectivity, like *gpsA* and *cdr*, but the role in ticks has not been investigated [43]. A *sodA* deletion mutant has altered levels of NADH and NAD^+^, as well as ATP, but all were approximately twofold different from wild type [44]. Other NADH-dependent enzymes are present in *B*. *burgdorferi*, but many have not been studied or are presumed to be essential, and none have been shown to influence metabolite (NADH and G3P) levels to the degree observed in the Δ*gpsA* mutant.

### Increased round body formation

*B*. *burgdorferi* morphology can change from the signature flat wave to RBs *in vitro* in response to environmental conditions, including nutrient stress [18,27,28,45] and *in vivo* in the tick [29] to presumably aid in *B*. *burgdorferi* persistence. RBs are a condensed spherical form of *B*. *burgdorferi* within an outer membrane that are viable and transitory [18,27–29]. The stringent response intracellular second messenger (p)ppGpp and the alternative sigma factor RpoS have been shown to control RB formation, as disrupting these global transcriptional regulators increased RB formation [18,29]. Cellular physiology also appears to be important as mutation of CoADR increases RB formation [42] and levels of acetyl-CoA and CoA are elevated in the Δ*gpsA* mutant (Fig 5C). Our results showing a massive transition of the Δ*gpsA* mutant to RBs during nutrient stress identify another metabolic enzyme involved in this morphologic change. Disruption of the redox potential of the cell may be a crucial signal for RB formation as both GpsA and CoADR use NADH as a cofactor and NADH levels dramatically increase and viability/reductase activity decrease in the Δ*gpsA* mutant (Fig 4, 5, and 6). Notably, many of the genes that regulate RB formation, *rel*_Bbu_, *cdr* and *gpsA*, are also important for persistence in the tick. The RB is not simply an aberrant non-viable form as many RBs remain viable (Fig 4) and can revert back to flat wave morphological forms [18,29,45]. Thus, environmental stresses that significantly alter the redox potential of the cell could trigger RB formation to aid survival under harsh conditions, such as in the tick between blood meals, but the mechanism and benefits of this morphological transformation for *B*. *burgdorferi* remain to be determined.

### Compromised survival *in vitro* and *in vivo* during persistence in ticks

*B*. *burgdorferi* must adapt to the stress of depleted nutrients in the tick midgut as the blood meal is consumed following larval and nymphal feeding. We and others have used RPMI *in vitro* to mimic the nutrient stress experienced in the tick midgut between blood meals and have identified the Rel_Bbu_-mediated stringent response and its effector DksA as important for survival under these conditions [17,18]. Here we found that *gpsA*, while not necessary for growth in nutrient-rich medium, was crucial for survival during nutrient stress *in vitro*, suggesting that NADH levels and redox potential have a key role in adapting to nutrient limitation. Completely severing the G3PDH node in the Δ*gpsA*/Δ*glpD* double mutant partially restored NADH levels and rescued both the survival defect and reductase activity, indicating that the inability to shuttle DHAP to G3P for lipid biosynthesis was not the cause of cell death *in vitro*. Also, the metabolome of the Δ*glpD* mutant showed altered levels of both G3P and DHAP (S3 Fig) but this mutant was neither compromised for survival nor reductase activity, suggesting the changes in these metabolites were not involved in adapting to nutrient stress. Furthermore, the increased levels of NADH in the Δ*gpsA* mutant could have theoretically increased the resistance to peroxide, but that was not observed under our conditions (S2 Fig). Other redox enzymes using NAD^+^/NADH as a cofactor play a role in *B*. *burgdorferi* survival in response to environmental stresses, particularly oxidative stress. The CoADR mutant had a growth defect *in vitro* and was more susceptible to lipid peroxidation, but not reactive oxygen species, and a *sodA* mutant was also more susceptible to superoxide radicals [41–43,46]. Thus, oxidoreductase enzymes using NAD^+^/NADH as a cofactor likely play a variety of roles in adapting to external stresses encountered throughout the enzootic environment.

Because decreased levels of G3P, needed for lipid and lipoprotein biosynthesis, in the Δ*gpsA* mutant could account for the survival defect observed under nutrient stress, we added glycerol to RPMI in an attempt to chemically rescue the phenotype. Surprisingly, even in wild-type *B*. *burgdorferi*, glycerol was cytotoxic in nutrient stress media (Fig 3C). Furthermore, glycerol was cytotoxic to all strains except those lacking *glpD* (Δ*glpD*, Δ*gpsA*/Δ*glpD* and *gpsA*^+^/Δ*glpD*; Fig 3C), indicating that GlpD was necessary for glycerol-mediated cell death. The cytotoxicity of glycerol may be due the continued action of GlpD oxidizing G3P to feed glycolysis, which amplifies the deficit of NAD^+^. Even more unexpected was the finding that added GlcNAc produced the same *glpD*-dependent cell death phenotype (Fig 3D). Because there is no known shared metabolic pathway between transport or metabolism of GlcNAc and transport or metabolism of glycerol (until glycolysis), we hypothesize that cofactor dysregulation in nutrient-depleted media may cause cell death. Perhaps the addition of another nutrient to the media could rescue *B*. *burgdorferi* viability by restoring the metabolic balance, but these studies remain to be done.

*gpsA* is also crucial for persistence *in vivo* in the tick: *gpsA* mutants seem to have difficulty colonizing larvae, although the difference from wild type-infected larvae was not significant, and the mutants were severely compromised (1000–10,000-fold) for persistence in unfed nymphs and replication in fed nymphs (Fig 8). Again, the dysregulation of the redox potential and NADH levels may explain the persistence defect as similar pathways have been identified as important in other studies. CoADR and Dps/NapA/BicA involved in the oxidative stress response were both shown to function during persistence in feeding nymphs [42,47]. Glycerol metabolism has been shown to be important for persistence in ticks [12,13], as *glpD* mutants have a threefold survival defect in fed larvae [14] and ten- to fifteenfold survival defect following nymph feeding [13]. The degree of dysregulation of NADH/NAD^+^ levels correlates with the severity of the persistence defect, suggesting this could be driving the tick phenotype. Unlike our *in vitro* results, the persistence defect in ticks was not restored in the Δ*gpsA*/Δ*glpD* double mutant indicating that there are additional challenges in the tick environment that the double mutant is unable to overcome. The defect in persistence in the tick could also be explained by compromised lipid and lipoprotein production in the *gpsA* mutant strains. The biosynthetic lipid and lipoprotein pathways, including GlpF, GlpK, 1-acyl-G3P acyltransferase (*bb0037*), and two fatty acid CoA ligases (*bb0137* and *bb0593*), are induced in ticks compared to mammalian hosts, suggesting an important role in the vector [48]. Thus, disruption of G3P production in the *gpsA* mutants may stress the lipid/lipoprotein biosynthetic machinery resulting in decreased viability and persistence. Taken together, our studies and previous work highlight the importance of this metabolic node for persistence in the tick vector and infectivity in the vertebrate host.

## Materials and methods

### Ethics statement

All animal experiments were approved by the University of Montana Institutional Animal Care and Use committee and followed the *Guidelines for the Care and Use of Laboratory Animals* from the National Institutes of Health.

### *B*. *burgdorferi* strains and growth conditions

Low-passage *B*. *burgdorferi* B31-5A4 [49], and genetically manipulated derivatives were grown and maintained in Barbour-Stoenner-Kelly II (BSK) liquid medium, pH 7.6, containing 6% rabbit serum (RS) (Pel-Freez Biologicals) [26] without gelatin. Cultures were grown at 35°C to 5–9 × 10^7^ cells ml^-1^ for experiments. Cell were enumerated using a Petroff-Hausser cell counting chamber [50].

### *B*. *burgdorferi* strain construction

The parental strain B31-5A4, referred to as wild type, was used to construct all mutant strains. *E*. *coli* TOP10F′ grown in lysogeny broth [51] were used for cloning. To construct the *gpsA* null mutant strain the regions upstream and downstream of the *gpsA* gene were amplified by PCR using KOD polymerase (Novagen) and the primers gpsA_U886F/gpsA_41R+AatII+AgeI or gpsA_1051F+AatII/gpsA_D1924R+AgeI (S1 Table), respectively, and B31-5A4 genomic DNA as a template. PCR products were cloned into pCR2.1-TOPO (Invitrogen) and verified by DNA-sequencing. Using the artificially engineered AgeI and AatII restriction sites, the two cloned PCR products were digested with AatII and AgeI and ligated together at the junction of the AatII site. The streptomycin/spectinomycin resistant cassette with the *flgB* promoter from *B*. *burgdorferi* [52] and *trpL* terminator from *Bacillus subtilis* [53] (*flgBp*-*aadA*-*trpLt*) containing flanking AatII sites was placed between the upstream and downstream DNA segments. The plasmid containing the *aadA* cassette replacing *gpsA* was purified and linearized using the restriction enzyme AhdI before electroporating competent B31-5A4 cells. 24 h after transformation, cells were plated in liquid BSK + RS in 96-well plates and transformants selected with 50 μg ml^-1^ streptomycin [54]. Colonies resistant to streptomycin were screened for the absence of *gpsA* by PCR analysis using the primers gpsA_U87F and gpsA_D1174R (S1B Fig). To generate the *gpsA cis*-complemented strain, the entire *gpsA* ORF and promoter were amplified by PCR using the primers gpsA_U886F and gpsA_D1125R+AatII+AgeI to yield the upstream segment. PCR amplification using the primers gpsA_1051F+AatII and gpsA_D1924+AgeI were used to produce the downstream fragment. As described above, these segments were ligated together and a gene conferring resistance to kanamycin (*flgBp*-*aphI*-*trpLt*) inserted downstream of the *gpsA* gene. The resulting plasmid was purified, linearized, and electroporated into competent Δ*gpsA* mutant cells, which were plated as described above except that transformants were selected in 200 μg ml^-1^ kanamycin. Restoration of the *gpsA* gene in isolated clones was confirmed by PCR analyses using the primers gpsA_U87F and gpsA_D1174R (S1B Fig).

The *glpD* null mutant strain was constructed as described above for the *gpsA* null mutant except that the primers glpD_U1016F and glpD_10R+AatII+AgeI were used to make the upstream segment and glpD_1568F+AatII and glpD_D2523R+AgeI were used to make the downstream segment. The gene conferring gentamicin resistance (*flgBp*-*aacC1*-*trpLt*) was inserted and transformants selected in 50 μg ml^-1^ gentamicin. Clones resistant to gentamicin were screened by PCR for the absence of the *glpD* gene using the primers glpD_U56F and glpD_D1669R (S1D Fig). The *glpD cis*-complemented strain was constructed as described for the *gpsA* complement except the upstream and downstream segments were amplified by PCR using the primers glpD_U719F/glpD_D1613R+AatII+AgeI and glpD_D1611F+AatII and glpD_D2523R+AgeI, respectively (S1 Table). The *flgBp*-*aphI*-*trpLt I* gene was inserted downstream of the *glpD* gene and transformants selected in kanamycin and clones screened by PCR using the primers glpD_U56F and glpD_D1669R (S1C and S1D Fig and S1 Table). Competent Δ*gpsA* mutant cells were transformed with the construct described above and selected in gentamicin to generate the Δ*gpsA*/Δ*glpD* double mutant strain. The same strategy used to make the *gpsA* complemented strain was used to make the *gpsA* complement of the double Δ*gpsA*/Δ*glpD* mutant to yield the *gpsA*^+^/Δ*glpD* strain. Likewise, the *glpD* complement of the double Δ*gpsA*/Δ*glpD* mutant to generate the Δ*gpsA*/*glpD*^+^ strain was as described above. The *gpsA* and *glpD* double complement of the Δ*gpsA*/Δ*glpD* double mutant was made by transforming competent *gpsA*^+^/Δ*glpD* cells described above with a *glpD* complement construct described above except that a streptomycin resistance cassette (*flgBp*-*aadA*-*trpLt*) was used and transformants selected in streptomycin. Absence of the *gpsA* gene in the double Δ*gpsA*/Δ*glpD* strain and restoration of the *gpsA* gene in the *gpsA*^+^/Δ*glpD* and double complemented g*psA*^+^/*glpD*^+^ isolated clones was confirmed by PCR analyses using the primers gpsA_385F and gpsA_493R (S1F Fig). Absence of the *glpD* gene in the Δ*gpsA*/Δ*glpD* double mutant and restoration of the *glpD* gene in the Δ*gpsA*/*glpD*^+^ single complement and *gpsA*^+^/*glpD*^+^ double complement was confirmed by PCR using the primers glpD_1F+SacI and glpD_1267R+AatII (S1G Fig). All strains used for infectivity experiments were screened for the presence of plasmids important for murine infectivity and persistence in the tick [55].

### Heterologous expression of *B*. *burgdorferi gpsA* in *E*. *coli* and growth analyses

The *B*. *burgdorferi gpsA* gene was amplified by PCR using KOD polymerase and the primers gpsA_1F+SacI and gpsA_1092R+PstI (S1 Table). The PCR product was cloned into pCR2.1-TOPO (Invitrogen) and verified by DNA sequencing. The *gpsA* gene was inserted into the multiple cloning site of the expression vector pUC18 by digesting both with the restriction enzymes PstI and SacI followed by ligation. The resulting pUC18-*gpsA*_Bb_ or the empty vector pUC18 were transformed into a *gpsA* null mutant *E*. *coli* strain (CGSC # 5424, *E*. *coli* Genetic Stock Center, Yale University) [25]. The *E*. *coli* strains Δ*gpsA*, Δ*gpsA* + pUC18 and Δ*gpsA* + pUC18-*gpsA*_Bb_ were grown in M9 minimal salts media containing 1% casamino acids with or without 1% glucose. To induce *gpsA*_Bb_ expression, 0.1 mM IPTG was added to all cultures. Cultures were grown in 96-well plates at 37°C with constant shaking in a BioTek Synergy HT plate reader (Agilent) where the OD_600_ was measured every 17 min.

### Nutrient stress and semi-solid BSK plating assay

*B*. *burgdorferi* strains were grown to 4–9 × 10^7^ cells ml^-1^, a portion diluted in BSK (approximately 200 spirochetes), and plated in semi-solid BSK (0 h time point). The remaining cultures were collected by centrifugation at 8000 x *g* for 10 min at 4°C. Cells were resuspended in RPMI 1640 without L-glutamine (Corning) either without or with 0.4% glycerol (Fisher Scientific) or 0.4% *N*-acetylglucosamine (MP Biomedicals) for nutrient stress assays as previously described [18]. After 24 h at 35°C cells, were diluted in BSK and plated in semi-solid BSK (24 h time points) and all plates (0 h and 24 h) allowed to grow at 35°C for about 14 days in a 5% CO_2_ incubator before colony enumeration.

### RedoxSensor staining and microscopy

*B*. *burgdorferi* strains were grown in BSK + RS at 35°C to ~7 × 10^7^ cells ml^-1^ before collection at 8,000 x *g* for 10 min at 4°C. Cell pellets were resuspended in warm RPMI and incubated at 35°C for 16 h. Cultures were then stained with *Bac*Light RedoxSensor Green and propidium iodide according to the manufacturer’s instructions (1 μl RedoxSensor Green and 1 μl propidium iodide in 1.0 ml culture) from the *Bac*Light RedoxSensor Green Vitality kit (Invitrogen). Cells were stained for 10 min at 35°C, collected by centrifugation (13,000 x *g*, 5 min, 4°C) and cell pellets resuspended in 1.0 ml PBS. 10 μl of cells were wet-mounted on a slide and live cells imaged using an Olympus BX51 fluorescence microscope with 100x/1.30 NA objective. Images were processed using ImageJ (National Institutes of Health; http://rsbweb.nih.gov/ij/) and Pixelmator (Pixelmator Team, Ltd). The mean percent cells stained with RedoxSensor Green and mean percent round body (RB) cells were determined from three independent biological replicates where at least 100 cells were enumerated for each strain. Significant difference between the means was determined by one-way ANOVA with a Tukey’s *post-hoc* test.

### Metabolomic analyses

For all liquid chromatography-mass spectrometry (LCMS) methods, LCMS grade solvents were used. Tributylamine and all synthetic molecular references were purchased from Millipore Sigma. LCMS grade water, methanol, isopropanol and acetic acid were purchased through Fisher Scientific.

*B*. *burgdorferi* strains were grown in BSK + RS at 35°C to ~3 × 10^7^ cells ml^-1^ before collection at 8,000 x *g* for 10 min at 4°C. Cells were washed twice with HEPES-NaCl buffer and pellets were flash frozen in dry-ice and ethanol and stored at -80°C until processing. To process cells for metabolomic analysis, cell pellets were thawed on ice, resuspended in 150 μL ice-cold methanol (Sigma) and then incubated at room temperature for 10 min. Following incubation an equal volume of LCMS grade water was added and the samples were vigorously vortexed, then centrifuged at 13,000 x *g* for 15 min. Supernatants were collected, filtered in a 0.2 μM nitrocellulose syringe filter (GE Healthcare), and then diluted 1:3 before analysis. All samples were separated using a SCIEX ExionLC AC system and measured using a SCIEX 5500 QTRAP mass spectrometer. Polar metabolites were analyzed using a previously established ion pairing method with modification [56,57]. Quality control samples were injected after every 10 injections to control for signal stability. Samples were separated with a Waters Atlantis T3 column (100Å, 3 μm, 3 mm X 100 mm) using a binary gradient from 5 mM tributylamine, 5 mM acetic acid in 2% isopropanol, 5% methanol, 93% water (v/v) to 100% isopropanol over 15 minutes. Each metabolite was identified and measured with two ion fragmentation pairs and a defined retention time.

All signals were integrated using MultiQuant Software 3.0.3. Signals with greater than 50% missing values were discarded and remaining missing values were replaced with the lowest registered signal value. All signals with a QC coefficient of variance greater than 30% were discarded. Metabolites with multiple ion pairs were quantified with the signature that displayed the highest signal to noise. All filtered datasets normalized against the total signal sum for the injection prior to analysis. Single and multi-variate analyses were performed in MarkerView Software 1.3.1. All univariate comparisons were subjected to a Benjamini-Hochberg cut-off at a false discovery rate of 5%.

### NAD^+^/NADH assays

*B*. *burgdorferi* strains were grown at 35°C to ~5 × 10^7^ cells ml^-1^ before collection at 14,000 x *g* for 10 min at 4°C. Cell pellets were washed twice with 1.0 ml cold H-N buffer (50 mM HEPES, 50 mM NaCl, pH 7.6), 15,800 x *g*, 5 min at 4°C, in cryovials. The supernatant was discarded and cell pellet flash frozen in liquid N_2_ and stored at -80°C. Samples were solubilized and NAD^+^ and NADH levels measured separately using the NAD/NADH-Glo Assay Kit (Promega) according to the manufacturer’s instructions. Luminescence was measured using a BMG Labtech/CLARIOstar plate reader. The levels of NAD^+^ and NADH were normalized to cell number from three independent biological replicates. Significant difference between the means was determined by one-way ANOVA with a Tukey’s *post-hoc* test.

### Immunoblot analyses

Equivalent amounts of *B*. *burgdorferi* whole cell extracts were analyzed by SDS-PAGE using Novex 4–20% Tris-Glycine polyacrylamide gels (Invitrogen) and proteins transferred to PVDF Immobilon membranes (Millipore). Membranes were blocked overnight at 4°C in blocking buffer (also used to dilute antibodies), which consisted of dPBS + 0.5% Tween-20 + 4% dried milk + 1% goat serum. Membranes were probed with rabbit anti-OspC (1:1000), mouse anti-OspA antibodies (1,2500, CDC), mouse anti-FlaB (1,100, gift from Tom Schwan), rabbit anti-GpsA (1:1000) or rabbit anti-GlpD (1,1000) followed by goat anti-rabbit or goat anti-mouse HRP-linked antibodies (Bio-Rad Laboratories) (1,10,000). Anti-GpsA and anti-GlpD antibodies were produced by GenScript using *B*. *hermsii* peptides as antigens. Detection was done by chemiluminescence (Amersham ECL Prime, GE Healthcare) and visualized using an LAS-3000 Intelligent Dark Box (Fujifilm Medical Systems USA).

### Mouse infectivity by needle inoculation and tick transmission

C3H-HeJ mice (Charles River Laboratories) were intradermal injected with 1 × 10^4^ to 1 × 10^6^ *B*. *burgdorferi* cells grown at 35°C to cell densities of 5–9 × 10^7^ cells ml^-1^. Infection was determined by culturing mouse ear biopsies in BSK II containing 50 μg ml^-1^ rifampicin, 20 μg ml^-1^ phosphomycin and 2.5 μg ml^-1^ amphotericin B and examining cultures by dark-field microscopy for the presence of spirochetes for 14 days. Five weeks post-infection, mice were sacrificed and ear, ankle and bladder tissues were collected, cultured and examined for spirochetes. Uninfected *Ixodes scapularis* larvae (National Tick Research and Education Resource, Oklahoma State University) were maintained in a 98% humidified chamber. To allow ticks to acquire *B*. *burgdorferi*, approximately 100 larvae per mouse were allowed to feed to repletion. Spirochete loads per tick were quantified by qPCR as described below. After infected larvae molted into nymphs (about 8 weeks), three mice were infested with three nymphs each and allowed to feed to repletion. Murine transmission was monitored by culturing ear, ankle and bladder tissues as described above. Persistence of *B*. *burgdorferi* in ticks was followed by qPCR as described below.

### Quantification of *B*. *burgdorferi* in ticks

Spirochete burdens in artificially infected ticks were assessed in fed larvae (one week, in groups of five), unfed nymphs (in groups of five) and fed nymphs (one week post feeding to repletion). DNA was extracted from ticks using the DNeasy Blood/Tissue kit (Qiagen) and the number of *B*. *burgdorferi* genomes in each tick determined by TaqMan qPCR with primers flaB_425F and flaB_542R and the flaB probe to the *flaB* gene (S1 Table) were 1 copy of *flaB* = one genome as previously described [18].

## Supporting information

S1 Table Oligonucleotides and probes used in this study. (DOCX)

S2 Table Metabolomic Analyses. (XLSX)

S1 Fig Strain construction and analyses. (A) Illustration of the *gpsA* mutant (Δ*gpsA*) and *gpsA* complemented (*gpsA*^+^) strains constructed by homologous recombination. The *gpsA* gene (*bb0368*) was replaced with the *B*. *burgdorferi* promoter from *flgB* fused to *aadA*, a gene conferring resistance to streptomycin (strep^R^). A wild-type copy of *gpsA* was reintroduced in *cis* using the *B*. *burgdorferi* promoter from *flgB* fused to *aphI*, a gene conferring resistance to kanamycin (kan^R^) to yield the *gpsA*^+^ strain. (B) Genomic DNA isolated from wild-type (WT), Δ*gpsA* and *gpsA*^+^ strains, and a no template control (NTC) was analyzed by PCR using primers 1 (gpsA_U87F) and 2 (gpsA_D1174R). (C) Illustration of the *glpD* mutant (Δ*glpD*) and *glpD* complemented (*glpD*^+^) strains constructed by homologous recombination. The *glpD* gene (*bb0243*) was replaced with the *B*. *burgdorferi* promoter from *flgB* fused to *aacC1*, a gene conferring resistance to gentamicin (gent^R^). A wild-type copy of *glpD* was reintroduced in *cis* using the *B*. *burgdorferi* promoter from *flgB* fused to *aadA*, a gene conferring resistance to streptomycin (strep^R^) to yield the *glpD*^+^ strain. (D) Genomic DNA isolated from WT, Δ*glpD* and *glpD*^+^ strains, and a NTC was analyzed by PCR using primers 3 (glpD_U56F) and 4 (glpD_D1669R). (E) Illustration of the double *gpsA*/*glpD* mutant (Δ*gpsA/*Δ*glpD*) and single and double complemented strains constructed by homologous recombination as described above. The *gpsA* complement of the double mutant (*gpsA*^+^/Δ*glpD*) was used to construct the *gpsA*-*glpD* double complement (*gpsA*^+^/*glpD*^+^). (F) Genomic DNA isolated from WT, the double *gpsA*/*glpD* mutant (Δ*gpsA*/Δ*glpD*), the *gpsA* complement of the double mutant (*gpsA*^+^/Δ*glpD*) and the *gpsA* and *glpD* double complement of the double mutant (*gpsA*^+^/*glpD*^+^) strains, and a NTC was analyzed by PCR using primers 5 (gpsA_385F) and 6 (gpsA_493R). (G) Genomic DNA isolated from WT, the double *gpsA*/*glpD* mutant (Δ*gpsA*/Δ*glpD*), the *glpD* complement of the double mutant (Δ*gpsA*/*glpD*^+^) and the *gpsA* and *glpD* double complement of the double mutant (*gpsA*^+^/*glpD*^+^) strains, and a NTC was analyzed by PCR using primers 7 (glpD_1F+SacI) and 8 (glpD_1267R+AatII). *B*. *burgdorferi* strains were grown in BSK+RS at 35°C, total cell lysates were collected and equal amounts separated by SDS-PAGE; proteins were transferred to membranes and analyzed by immunoblot with antibodies against (H) GpsA (upper panel) or FlaB (lower panel) or (I) GlpD (upper panel) or FlaB (lower panel).
(TIF)

S2 Fig Sensitivity of the *gpsA* mutant to reactive oxygen species (H_2_O_2_). Wild-type (WT), *gpsA* mutant and *gpsA* complemented (*gpsA*^+^) strains were grown in pyruvate-free BSK + RS at 35°C to late log phase. Cultures were left untreated or treated with freshly prepared 0.2 mM H_2_O_2_ for 2 h at 35°C before plating in semi-solid BSK. Plates were incubated for 10–15 days at 35°C, individual colonies were enumerated and percent survival expressed as (# of colonies in 0.2 mM H_2_O_2_ / # colonies in untreated) × 100. Data are from three biological replicates where circles represent individual data points and the bar represents the mean. No statistical difference was observed between the mean survival of each strain as determined by one-way ANOVA with a Tukey’s *post-*hoc test.
(TIF)

S3 Fig GlpD deletion results in an opposing but less severe metabolic pattern to GpsA deletion. (A) Metabolites that significantly vary between Δ*glpD* and wild type (WT) with a false discovery rate (FDR) less than 5%. Values in the heatmap at left are displayed as the log_2_(fold change mutant versus WT) and values in the accompanying heatmap at right indicate whether that metabolite passes a 5% FDR filter for the indicated comparison as assessed by a Benjamini-Hochberg correction. (B) Metabolic map of the changes in glycolysis and the glycerol shunt that occur with the loss of GlpD. All measured metabolites in the included pathways are displayed. The log_2_(fold change Δ*glpD* versus WT) is displayed as color of the node and the -log(*p*-value) is displayed as the size of the node. Enzymes in the glycerol arm of metabolism are displayed as diamonds.
(TIF)

S4 Fig GpsA does not affect OspC and OspA protein levels. Extracts from wild-type (WT), Δ*gpsA* mutant, *gpsA* complement (*gpsA*^+^) and Δ*gpsA*/Δ*glpD* double mutant cells grown at 35°C (without a temperature shift) were separated by SDS-PAGE and analyzed by immunoblot using antibodies against FlaB, OspC or OspA. At least three independent experiments were performed and representative image is shown.
(TIF)
