# Supplementary material for: The glycerol-3-phosphate dehydrogenases GpsA and GlpD constitute the oxidoreductive metabolic linchpin for Lyme disease spirochete host infectivity and persistence in the tick
Source: PLoS Pathog. 2022 Mar 7;18(3):e1010385. doi: 10.1371/journal.ppat.1010385 (PMC8929704; doi:10.1371/journal.ppat.1010385)
Supplement: S1 Table — (DOCX) [file ppat.1010385.s001.docx]

Table S1. Oligonucleotides and probes used in this study.

| Primer name | Sequence (5′ to 3′) |
| --- | --- |
| glpD_U1016F | TCTTGAAGGAAGTGTTTTTA |
| glpD_10R +AatII +AgeI | **ACCGGT**AAT**GACGTC**ATTCCTCCATTTTGGTTAAT |
| glpD_1568F + AatII | **GACGTC**AAAAATATTTAATTTAAAATTAATTTTT |
| glpD_D2523R +AgeI | **ACCGGT**GAGAGTGGTAGAGTTAAATC |
| glpD_U719F | CCAACTTTGAAATTCAAGAA |
| glpD_D1631R+Aa+Ag | **ACCGGT**TTT**GACGTC**TAAAAGGGATTGCTTAACAA |
| glpD_D1611F+AatII | **GACGTC**ATTGTTAAGCAATCCCTTT |
| glpD_D2523R+AgeI | **ACCGGT**GAGAGTGGTAGAGTTAAATC |
| glpD_1F+SacI | **GAGCTC**ATGGAGGAATATTTAAATTTCA |
| glpD_1267R+AatII | **GACGTC**TGCTTCTTCTTTTTTAAGGT |
| glpD_U56F | GTCTTTTCGAATTTCTTCAA |
| glpD_D1669R | TTGGGTGTAAGACTTTTTAA |
| gpsA_1F+SacI | **GAGCTC**ATGAGTTTTTATAAGGTTATAGG |
| gpsA_1092R+PstI | **CTGCAG**TTATTGTCTAACATCTCTCA |
| gpsA_U886F | TGATGGTGAATTGCTAATTA |
| gpsA_41R+AatII+AgeI | **ACCGGT**ATT**GACGTC**ATTTTCATAAATACTCCCCC |
| gpsA_1051F+AatII | **GACGTC**CCAAATCTGTTATTGAGTA |
| gpsA_D1924R+AgeI | **ACCGGT**AAAGAAGAAATTGGTTCAAC |
| gpsA_U886F | TGATGGTGAATTGCTAATTA |
| gpsA_D1125R+AatII+AgeI | **ACCGGT**TGA**GACGTC**AAAAACAATAAAGCCTCTG |
| gpsA_1051F+AatII | **GACGTC**CCAAATCTGTTATTGAGTA |
| gpsA_D1924R +AgeI | **ACCGGT**AAAGAAGAAATTGGTTCAAC |
| gpsA_U87F | TTGGGAAAGCTTTTAATAGA |
| gpsA_D1174R | TGAAATAATCGAGGATGTTT |
| gpsA_385F | ACTCAGACAGTTATTGAAGCTGCTGA |
| gpsA_493R | TTATCACACCAAGCCCAACTTCCTC |
| flaB_423F | TTCTCAAAATGTAAGAACAGCTGAAGA |
| flaB_542R | TGGTTTGTCCAACATGAACTC |
| flaB probe | 6-FAM-TCACTTTCAGGGTCTCAAGCGTCTTGGAC-TAMRA |
