# Supplementary figures and images for: The glycerol-3-phosphate dehydrogenases GpsA and GlpD constitute the oxidoreductive metabolic linchpin for Lyme disease spirochete host infectivity and persistence in the tick
Source: PLoS Pathog. 2022 Mar 7;18(3):e1010385. doi: 10.1371/journal.ppat.1010385 (PMC8929704; doi:10.1371/journal.ppat.1010385)

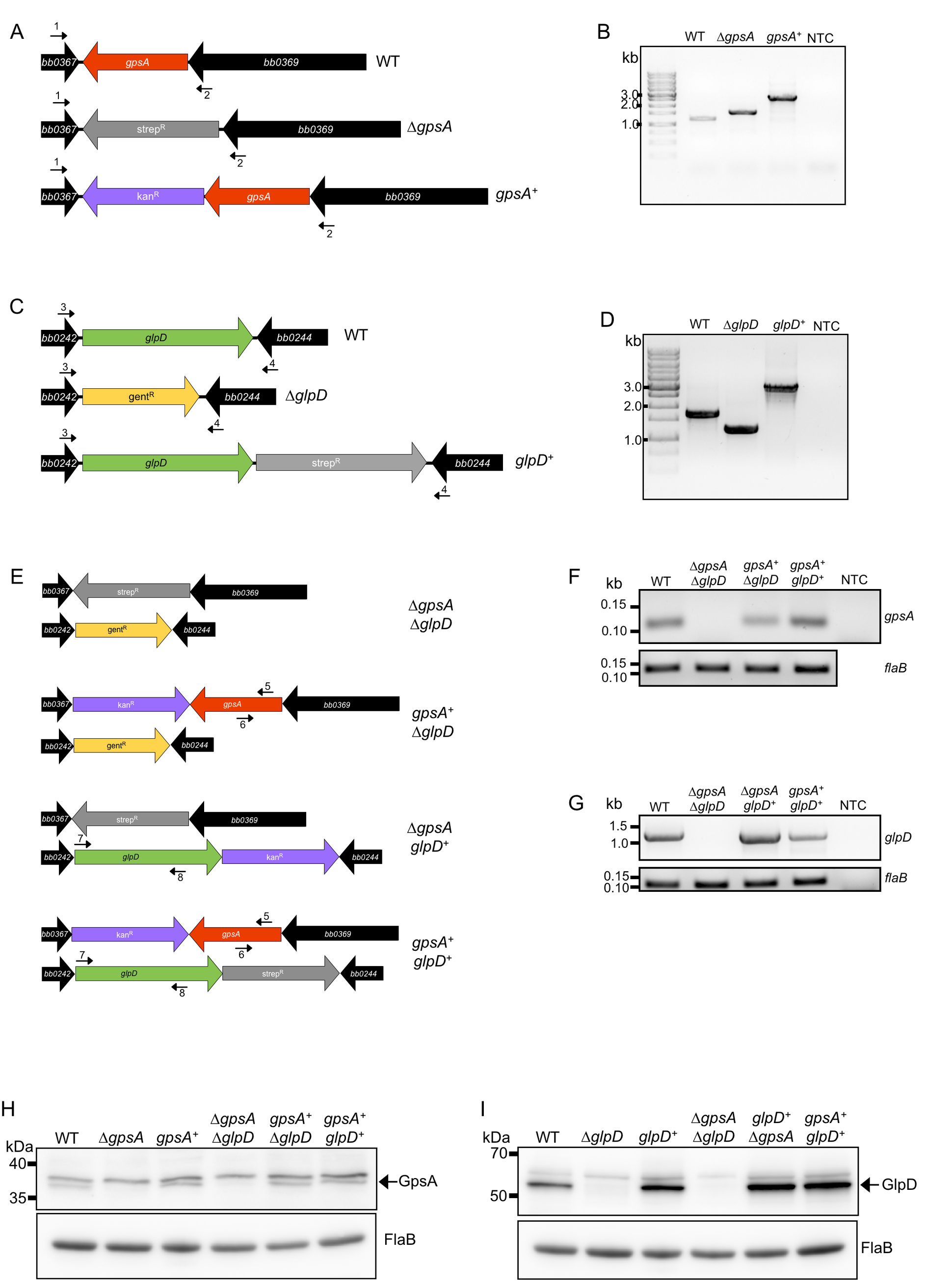

Supplement: S1 Fig — (A) Illustration of the gpsA mutant (ΔgpsA) and gpsA complemented (gpsA+) strains constructed by homologous recombination. The gpsA gene (bb0368) was replaced with the B. burgdorferi promoter from flgB fused to aadA, a gene conferring resistance to streptomycin (strepR). A wild-type copy of gpsA was reintroduced in cis using the B. burgdorferi promoter from flgB fused to aphI, a gene conferring resistance to kanamycin (kanR) to yield the gpsA+ strain. (B) Genomic DNA isolated from wild-type (WT), ΔgpsA and gpsA+ strains, and a no template control (NTC) was analyzed by PCR using primers 1 (gpsA_U87F) and 2 (gpsA_D1174R). (C) Illustration of the glpD mutant (ΔglpD) and glpD complemented (glpD+) strains constructed by homologous recombination. The glpD gene (bb0243) was replaced with the B. burgdorferi promoter from flgB fused to aacC1, a gene conferring resistance to gentamicin (gentR). A wild-type copy of glpD was reintroduced in cis using the B. burgdorferi promoter from flgB fused to aadA, a gene conferring resistance to streptomycin (strepR) to yield the glpD+ strain. (D) Genomic DNA isolated from WT, ΔglpD and glpD+ strains, and a NTC was analyzed by PCR using primers 3 (glpD_U56F) and 4 (glpD_D1669R). (E) Illustration of the double gpsA/glpD mutant (ΔgpsA/ΔglpD) and single and double complemented strains constructed by homologous recombination as described above. The gpsA complement of the double mutant (gpsA+/ΔglpD) was used to construct the gpsA-glpD double complement (gpsA+/glpD+). (F) Genomic DNA isolated from WT, the double gpsA/glpD mutant (ΔgpsA/ΔglpD), the gpsA complement of the double mutant (gpsA+/ΔglpD) and the gpsA and glpD double complement of the double mutant (gpsA+/glpD+) strains, and a NTC was analyzed by PCR using primers 5 (gpsA_385F) and 6 (gpsA_493R). (G) Genomic DNA isolated from WT, the double gpsA/glpD mutant (ΔgpsA/ΔglpD), the glpD complement of the double mutant (ΔgpsA/glpD+) and the gpsA and glpD double complement of the double mutant [file ppat.1010385.s003.tif]

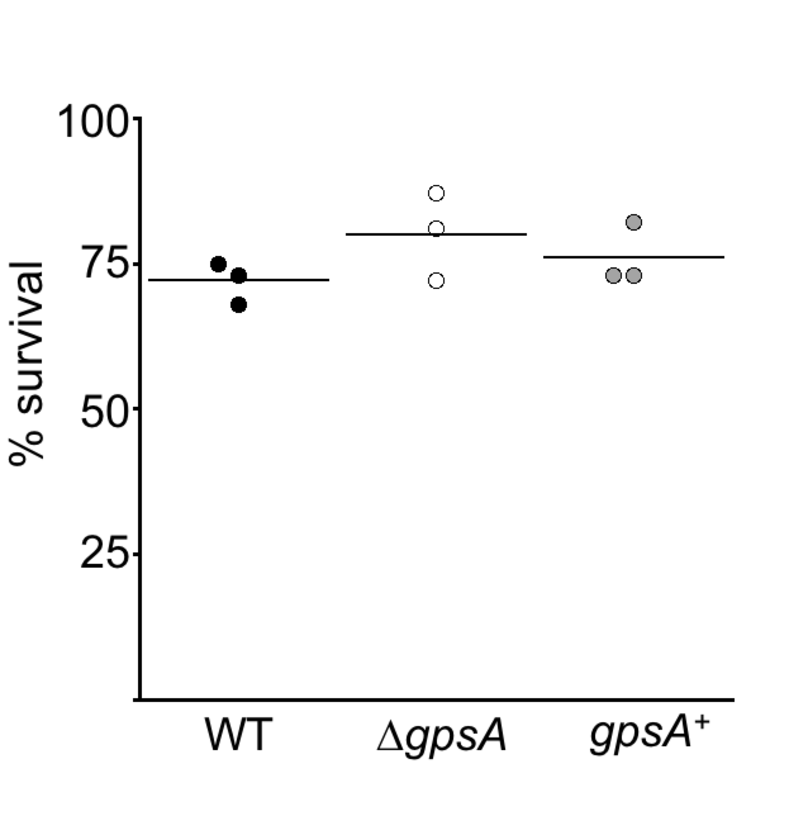

Supplement: S2 Fig — Wild-type (WT), gpsA mutant and gpsA complemented (gpsA+) strains were grown in pyruvate-free BSK + RS at 35°C to late log phase. Cultures were left untreated or treated with freshly prepared 0.2 mM H2O2 for 2 h at 35°C before plating in semi-solid BSK. Plates were incubated for 10–15 days at 35°C, individual colonies were enumerated and percent survival expressed as (# of colonies in 0.2 mM H2O2 / # colonies in untreated) × 100. Data are from three biological replicates where circles represent individual data points and the bar represents the mean. No statistical difference was observed between the mean survival of each strain as determined by one-way ANOVA with a Tukey’s post-hoc test. (TIF) [file ppat.1010385.s004.tif]

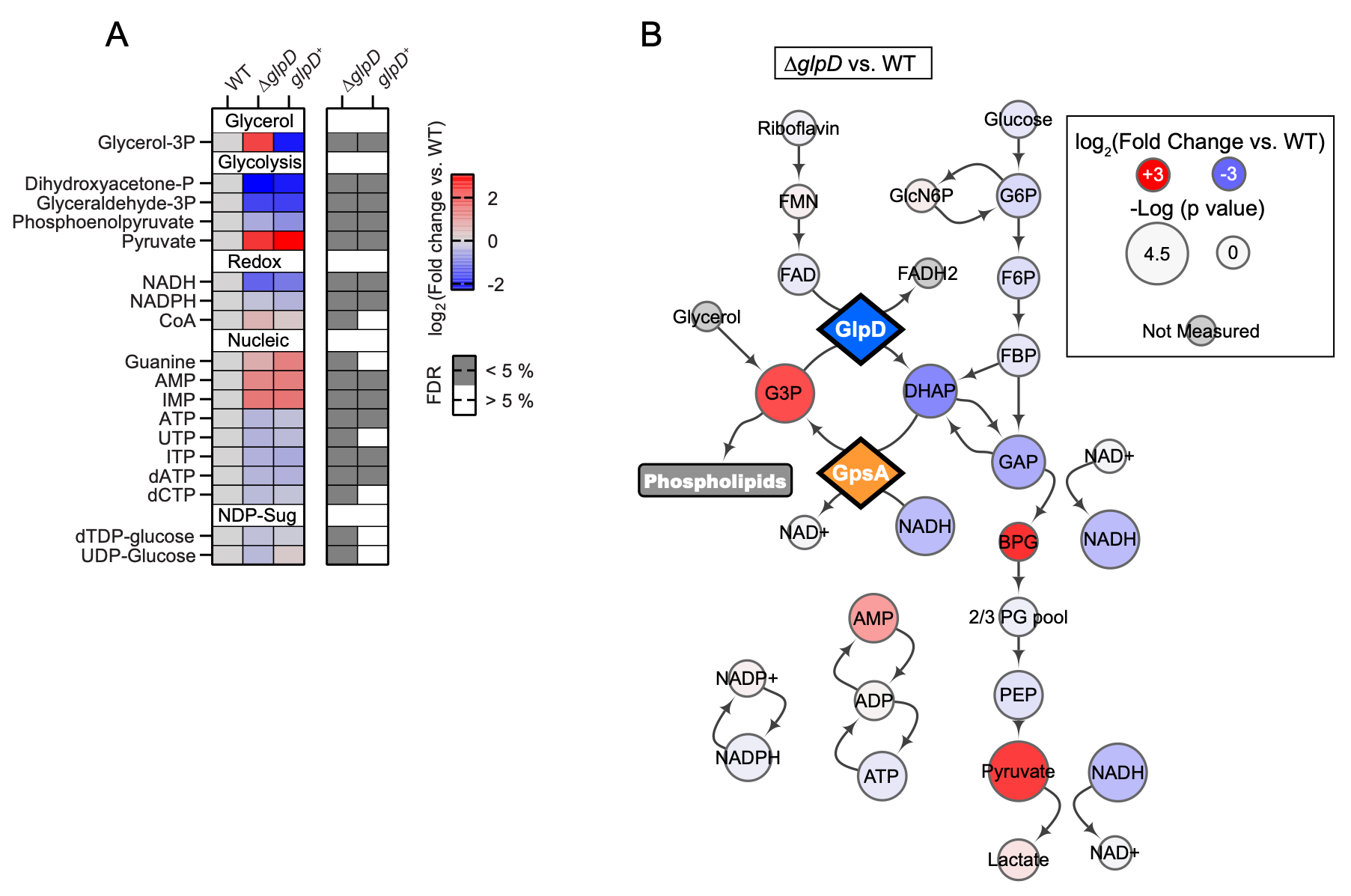

Supplement: S3 Fig — (A) Metabolites that significantly vary between ΔglpD and wild type (WT) with a false discovery rate (FDR) less than 5%. Values in the heatmap at left are displayed as the log2(fold change mutant versus WT) and values in the accompanying heatmap at right indicate whether that metabolite passes a 5% FDR filter for the indicated comparison as assessed by a Benjamini-Hochberg correction. (B) Metabolic map of the changes in glycolysis and the glycerol shunt that occur with the loss of GlpD. All measured metabolites in the included pathways are displayed. The log2(fold change ΔglpD versus WT) is displayed as color of the node and the -log(p-value) is displayed as the size of the node. Enzymes in the glycerol arm of metabolism are displayed as diamonds. (TIF) [file ppat.1010385.s005.tif]

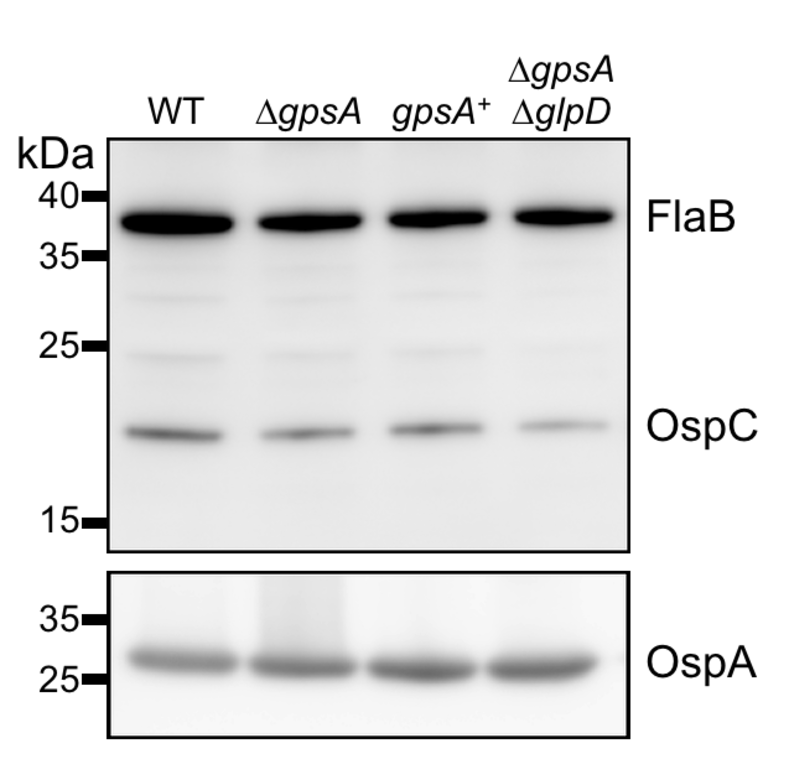

Supplement: S4 Fig — Extracts from wild-type (WT), ΔgpsA mutant, gpsA complement (gpsA+) and ΔgpsA/ΔglpD double mutant cells grown at 35°C (without a temperature shift) were separated by SDS-PAGE and analyzed by immunoblot using antibodies against FlaB, OspC or OspA. At least three independent experiments were performed and representative image is shown. (TIF) [file ppat.1010385.s006.tif]
